# Risk-stratified surveillance after LEEP: a nomogram integrating HPV persistence, margin status, and clinical factors to predict CIN2+ recurrence

**DOI:** 10.3389/fonc.2026.1786335

**Published:** 2026-03-03

**Authors:** Haixia Shang, Xiaofeng Shi, Hongxin Yu, Yaqian Feng, Yue Huang, Nan Guo, Xin Guo, Qi Guo, Xiaoxue Wang, Jingfen Sun

**Affiliations:** 1 Department of Obstetrics and Gynecology, Shanxi Bethune Hospital, Shanxi Academy of Medical Sciences, Third Hospital of Shanxi Medical University, Tongji Shanxi Hospital, Taiyuan, China; 2 Department of Obstetrics and Gynecology, Third Hospital of Shanxi Medical University, Shanxi Bethune Hospital, Shanxi Academy of Medical Sciences, Tongji Shanxi Hospital, Taiyuan, China

**Keywords:** cervical intraepithelial neoplasia, loop electrosurgical excision procedure, nomogram, persistent high-risk HPV, recurrent CIN2+, surgical margin status

## Abstract

**Background:**

Cervical intraepithelial neoplasia (CIN) recurrence after loop electrosurgical excision procedure (LEEP) remains a clinically consequential barrier to cervical cancer prevention, and risk stratification tools tailored to real-world practice are limited in China. This study developed and internally validated a clinical prediction nomogram for histologically confirmed CIN2+ recurrence after LEEP.

**Methods:**

A retrospective single-center cohort was assembled of women treated with LEEP for CIN2+ between January 2018 and October 2024. Candidate predictors included demographic and reproductive factors, smoking, HPV vaccination, prior cervical treatment, transformation zone type, LEEP pathology (including adenocarcinoma *in situ* [AIS] and margin status), pre-/post-treatment high-risk HPV measures, and neutrophil-to-lymphocyte ratio (NLR). Time-to-recurrence was analyzed using Cox regression with hierarchical domain modeling. A nomogram was constructed from the final multivariable model and evaluated for discrimination and calibration.

**Results:**

Among 2,230 women (median follow-up 31.8 months, IQR 19.6–43.5), 334 developed CIN2+ recurrence (15.0%), with a median time to recurrence of 15.6 months (IQR 8.2–24.3). Persistent HPV infection occurred in 50.6% of women with recurrence versus 23.1% without recurrence (*p* < 0.001). Persistent HPV infection (same genotype pre-/post-LEEP) was the strongest independent predictor (adjusted hazard ratio [aHR] 2.51, 95% CI 1.99–3.16). Additional independent predictors included unvaccinated status (aHR 1.54, 95% CI 1.08–2.20), multiple positive margins (aHR 1.52, 95% CI 1.08–2.14), AIS versus CIN2 (aHR 1.48, 95% CI 1.03–2.12), prior cervical treatment (aHR 1.38, 95% CI 1.04–1.84), single positive margin (aHR 1.38, 95% CI 1.02–1.87), and higher NLR (per one-unit increase: aHR 1.21, 95% CI 1.02–1.44). Model discrimination increased across hierarchical models from 0.516 (model 1) and 0.562 (model 3) to 0.619 in the final model. Risk stratification separated low-, intermediate-, and high-risk groups with observed 24-month recurrence rates of 6.2%, 14.8%, and 31.5%, respectively (*p* for trend <0.001).

**Conclusion:**

In a contemporary Chinese single-center cohort, genotype-defined persistent HPV infection and margin burden were dominant determinants of CIN2+ recurrence after LEEP, with vaccination status and NLR providing additional stratification. The resulting nomogram offers a pragmatic framework for risk-adapted surveillance, pending external multicenter validation.

## 1 Introduction

Cervical cancer remains a major global public health challenge, ranking as the fourth most common malignancy among women worldwide, with an estimated 662,044 new cases and 348,709 deaths reported in 2022 (1, 2). The burden of this largely preventable disease exhibits striking geographical disparities, with approximately 90% of new cases and deaths occurring in low- and middle-income countries (3). Despite significant advances in prevention and screening strategies, the Global Cancer Observatory 2020 estimates revealed that 172 of 185 countries exceeded the World Health Organization’s cervical cancer elimination threshold of four cases per 100,000 women-years, underscoring the substantial gap between current disease burden and elimination targets (4). The persistent human papillomavirus (HPV) infection, particularly with high-risk genotypes, accounts for virtually all cervical cancer cases, with HPV types 16 and 18 alone responsible for approximately 70% of cervical cancers globally (5). Recent projections indicate that without scaled-up interventions, the global burden of cervical cancer is expected to increase by 56.8% in incidence and 80.7% in mortality by 2050, with early-onset cervical cancer showing particularly alarming upward trends in transitioning countries (6, 7).

The loop electrosurgical excision procedure (LEEP) has emerged as the primary treatment modality for high-grade cervical intraepithelial neoplasia (CIN2/3), offering several advantages over traditional cold knife conization, including shorter operative time, lower cost, and equivalent therapeutic efficacy (8, 9). Extensive international evidence demonstrates LEEP’s effectiveness, with cure rates ranging from 73% to 99% across diverse populations (10, 11). However, despite optimal treatment, post-LEEP recurrence of CIN2+ lesions remains a significant clinical concern, with reported recurrence rates varying from 6.1% to 21.9% across different studies (12, 13). Multiple risk factors have been consistently identified as predictors of recurrence in international literature, including positive surgical margins, persistent HPV infection, smoking status, immunosuppression, and specific high-risk HPV genotypes, particularly HPV 16, 18, and 33 (14, 15). Post-treatment HPV persistence emerges as the most robust predictor, with studies demonstrating hazard ratios exceeding 2.8 for recurrence risk and approximately 80% of recurrences occurring within the first 2 years after LEEP (16, 17). The critical role of post-treatment HPV testing has been emphasized by recent investigations showing that HPV 16 persistence confers a sevenfold increased risk of CIN2+ recurrence, with most relapses manifesting within 18–24 months of persistent infection (18). Additionally, emerging evidence highlights the significance of inflammatory biomarkers, with the neutrophil-to-lymphocyte ratio demonstrating independent predictive value for post-LEEP recurrence (19).

China contributes disproportionately to the global cervical cancer burden, accounting for approximately 23% of new cases and 16% of deaths worldwide in 2022 (6, 7). The age-standardized incidence and mortality rates in China are 10.42 and 2.84 per 100,000 women, respectively, with concerning trends showing an 8.5% annual increase in incidence during 2000–2016 (20). Despite being home to one-fifth of the world’s cervical cancer cases, China faces unique challenges in disease prevention and control. HPV vaccination coverage remains critically low, with less than 1% of girls aged 9–14 years vaccinated as of 2020, primarily due to delayed vaccine availability (approved only in 2016), high costs, and absence of national immunization policies (21, 22). Furthermore, cervical cancer screening coverage in China stands at only 33%, substantially below the global average of 36% and far from the WHO 90–70–90 elimination targets (21, 23). Marked urban–rural disparities compound these challenges, with lifetime screening coverage of 41.1% in urban areas compared to 32.4% in rural regions (24). The late implementation of both HPV vaccination and organized screening programs has resulted in a substantial cohort of women who received neither primary nor secondary prevention, placing them at elevated risk for cervical neoplasia and necessitating comprehensive follow-up strategies post-treatment (24, 25). In the Chinese context, specific factors such as lower HPV vaccination rates, different healthcare access patterns, and variations in high-risk HPV genotype distribution (notably higher prevalence of HPV 52 and 58) may influence the recurrence patterns and risk stratification models developed in Western populations (26, 27).

Despite the extensive literature on CIN recurrence after LEEP, significant research gaps persist both globally and within the Chinese healthcare context (12). While multiple studies have identified individual risk factors for recurrence, comprehensive prediction models integrating multiple clinical, pathological, and molecular variables remain limited (12, 28, 29). Existing nomograms and machine learning models have shown promising results, with C-indices ranging from 0.619 to 0.975, yet most have been developed and validated in Western populations (30, 31). The applicability and performance of these models in the Chinese population remain uncertain, given the distinct epidemiological landscape, including differential HPV genotype distribution, lower vaccination coverage, and unique healthcare system characteristics. Critically, there is a paucity of externally validated, user-friendly clinical prediction tools specifically tailored for Chinese women undergoing LEEP for high-grade CIN (32, 33). Most Chinese studies have focused on residual disease rather than long-term recurrence risk, and comprehensive models incorporating post-treatment HPV persistence, vaccination status, lifestyle factors, and inflammatory biomarkers within a single predictive framework are notably absent (34, 35). Furthermore, the optimal risk stratification thresholds and their clinical utility in guiding individualized surveillance strategies remain inadequately defined for the Chinese healthcare setting, where resource allocation and follow-up protocols differ substantially from high-income countries (36, 37).

The present study aims to address these critical knowledge gaps by developing and internally validating a comprehensive clinical nomogram for predicting CIN2+ recurrence following LEEP in a Chinese population. Specifically, we sought to identify independent predictors of recurrence through multivariable Cox proportional hazards modeling, construct a clinically applicable nomogram integrating demographic, clinical, pathological, and molecular variables, and evaluate the model’s discriminative ability and calibration performance to facilitate risk-stratified post-LEEP surveillance strategies.

## 2 Methods

### 2.1 Study design and population

This single-center retrospective cohort study was conducted at the Department of Obstetrics and Gynecology, Shanxi Bethune Hospital, Shanxi Academy of Medical Sciences, Third Hospital of Shanxi Medical University, Tongji Shanxi Hospital, Taiyuan, China. We enrolled consecutive women who underwent loop electrosurgical excision procedure (LEEP) for histologically confirmed cervical intraepithelial neoplasia grade 2 or higher (CIN2+) between January 2018 and October 2024, with follow-up data extracted from electronic medical records through October 2025. This study was purely retrospective; all data were retrieved from existing hospital databases, and no participants were recruited or contacted for the purpose of this research. The study protocol adhered to the Strengthening the Reporting of Observational Studies in Epidemiology (STROBE) guidelines (38). The development and validation of the prediction model were reported in accordance with the Transparent Reporting of a multivariable prediction model for Individual Prognosis or Diagnosis (TRIPOD) statement (39).

The inclusion criteria were age ≥18 years at LEEP, histologically confirmed cervical intraepithelial neoplasia grade 2 or higher (CIN2+), including adenocarcinoma *in situ*, on index LEEP specimen reviewed by two independent pathologists, complete baseline clinical and pathological data, and minimum 12-month follow-up or until recurrence diagnosis. The exclusion criteria included previous hysterectomy, pregnancy at time of LEEP or within 6 months post-LEEP, invasive cervical cancer on index specimen (except microinvasive stage IA1 managed definitively with LEEP), HIV-positive serostatus, concurrent vaginal or vulvar high-grade neoplasia, documented loss to follow-up within 12 months, incomplete pathological specimens precluding margin assessment, or participation in investigational HPV therapeutic vaccine trials. The final cohort comprised 2,230 women with 334 recurrence events (15.0%) over median 31.8 months of follow-up (interquartile range 19.6–43.5 months).

### 2.2 Clinical procedures and follow-up

All patients underwent standardized pre-LEEP evaluation, including structured clinical history and physical examination with height/weight measurement, liquid-based cytology using ThinPrep system (Hologic, USA) classified per Bethesda 2014 (40), high-risk HPV DNA testing via Hybrid Capture 2 (HC2, Qiagen) detecting 13 oncogenic types collectively (16, 18, 31, 33, 35, 39, 45, 51, 52, 56, 58, 59, and 68) with positivity threshold RLU/CO ≥1.0, HPV genotyping via Linear Array (Roche) for HC2-positive cases, colposcopic examination per IFCPC 2011 nomenclature documenting transformation zone type and lesion characteristics, and colposcopy-directed punch biopsy with endocervical curettage when indicated (41). Complete blood count with differential was obtained within 1 week pre-LEEP using Sysmex XN-series automated analyzer; neutrophil-to-lymphocyte ratio (NLR) was calculated as absolute neutrophil count divided by absolute lymphocyte count. All pre-LEEP histopathology was reviewed by two independent gynecologic pathologists using standardized criteria; discordant cases were adjudicated by a senior pathologist. CIN2 diagnoses underwent p16 immunohistochemistry (CINtec, Roche) when histologically equivocal.

LEEP procedures were performed in outpatient setting under local anesthesia (paracervical block with 1% lidocaine plus epinephrine 1:100,000) using standardized electrosurgical settings (blended current 35–45 watts for cutting, pure coagulation 40–50 watts for hemostasis). Single-pass excision technique was attempted for all lesions to produce intact specimens optimal for margin assessment; multiple-pass approach was employed only when single-pass was inadequate due to lesion size (>2.5 cm), extensive circumferential involvement (>75%), or deep endocervical extension. Specimens were oriented with suture at 12 o’clock position, measured in three dimensions using calibrated calipers (transverse diameter, anteroposterior diameter, and cone depth in millimeters), and submitted fresh to pathology.

All LEEP specimens underwent standardized processing in our CAP-accredited pathology laboratory. The oriented specimens were fixed in 10% neutral buffered formalin for 12–24 h, sectioned perpendicular to the endocervical axis at 2- to 3-mm intervals, entirely embedded, and stained with hematoxylin–eosin. The pathologists evaluated the specimens using the standardized synoptic reporting type of documenting: specimen dimensions, highest-grade CIN lesion (CIN2, CIN3, or adenocarcinoma *in situ*), extent of dysplasia, glandular involvement (binary: CIN extension into endocervical gland crypts present versus absent), and three-margin status. The margins were assessed as endocervical margin (superior edge), ectocervical margin (inferior edge), and deep/stromal margin (lateral circumferential and basal). Each margin was classified as positive (CIN2+ at margin or within 1 mm) or negative (>1 mm clearance) based on the minimum distance measured via calibrated ocular micrometer on multiple sections. Multifocal margin involvement was categorized as none (all margins negative), single (one margin positive), or multiple (≥2 margins positive). All specimens received mandatory dual-pathologist review; final diagnoses were recorded in an electronic pathology system.

Post-LEEP surveillance followed institutional protocol adapted from ASCCP and Chinese guidelines: co-testing (cytology plus HPV) at 6–9, 12–15, 18–21, and 24–27 months post-LEEP, then annually through year 5 (42, 43). Colposcopies with directed biopsy was triggered by any cytological abnormality (ASC-US or higher), positive high-risk HPV regardless of cytology, or clinical concern. When colposcopy revealed no visible lesion despite positive co-testing, endocervical curettage was performed. All surveillance procedures were documented in the electronic tracking system with automated reminders for missed appointments and telephone contact attempts before classifying patients as lost to follow-up.

The primary outcome was histologically confirmed CIN2+ recurrence, defined as detection of CIN2, CIN3, adenocarcinoma *in situ*, or invasive cervical cancer on biopsy or excisional specimen obtained ≥6 months following index LEEP. This 6-month threshold distinguished true recurrence from residual/persistent disease. Recurrence required histological confirmation by two pathologists; cytological abnormalities alone were not considered recurrence. Follow-up duration was calculated from index LEEP to recurrence diagnosis, death from any cause, documented loss to follow-up, or study end (October 31, 2025). Electronic medical records were cross-referenced with regional cancer registry, pathology database, and civil death registry for outcome ascertainment.

### 2.3 Variable definitions and measurements

Demographic variables: Age at LEEP was calculated as completed years from birth to procedure date, analyzed continuously per 10-year increment and categorically (<40 versus ≥40 years). Body mass index (BMI) was calculated as weight (kg) ÷ height² (m²) from measurements obtained at pre-LEEP visit, analyzed continuously per five-unit increment and categorized per WHO Asian-Pacific criteria (underweight <18.5, normal 18.5–22.9, overweight 23.0–24.9, obese ≥25.0 kg/m²) (44). Menopausal status was classified as premenopausal (regular cycles within 12 months or irregular cycles with last period within 12 months and age <45 years) versus postmenopausal (≥12 months amenorrhea without alternative cause in women ≥45 years or bilateral oophorectomy regardless of age).

Reproductive history: Parity was defined as the number of pregnancies reaching ≥24 weeks of gestation resulting in live birth or stillbirth, categorized as nulliparous (0), one to two children, or ≥3 children. New sexual partner during follow-up was defined as self-reported acquisition of new partner between LEEP and recurrence/last visit. This information was retrieved from standard nursing assessment forms recorded in the electronic medical record, which are administered as part of routine clinical care at each surveillance visit (binary: yes/no).

Lifestyle factors: Smoking status was classified as never smoker (lifetime <100 cigarettes), former smoker (≥100 cigarettes but cessation >6 months pre-LEEP), or current smoker (active use within 6 months of LEEP or continued during follow-up), with former and current combined as “ever smoker” for analysis. HPV vaccination status was documented via immunization registry, vaccination card, or medical records, classified as unvaccinated (no doses), partially vaccinated (incomplete schedule), or fully vaccinated (completed age-appropriate regimen: three doses for bivalent/quadrivalent or two doses for nonavalent if age <15 years at initiation).

Pre-LEEP disease characteristics: Previous cervical treatment was defined as any documented prior therapeutic intervention (cryotherapy, laser, previous LEEP, and conization) identified through medical records (binary). Referral cytology was recorded as the most severe result within 6 months pre-LEEP (ASC-US, LSIL, HSIL, ASC-H, and AGC per Bethesda 2014). Pre-LEEP biopsy histology was documented as highest-grade diagnosis on punch biopsy/ECC within 3 months pre-LEEP (CIN1, CIN2, and CIN3). Transformation zone type was assessed colposcopically per IFCPC 2011: type 1 (squamocolumnar junction fully visible), type 2 (junction visible with endocervical component), or type 3 (junction not fully visible).

LEEP procedure variables: Indication was classified as diagnostic (biopsy-proven CIN2+ requiring LEEP for definitive assessment) versus therapeutic (see-and-treat or treatment following biopsy). Excision type was recorded as single-pass (entire lesion in one piece) versus multiple-pass (≥2 pieces due to size/extent). Cone depth was measured in millimeters by a pathologist on fresh specimen as the vertical dimension from the ectocervical surface to the deepest endocervical extent, analyzed continuously per 5-mm increment.

Pathological variables: Highest CIN grade in LEEP specimen was classified as CIN2 (moderate dysplasia), CIN3 (severe dysplasia/carcinoma *in situ*), or adenocarcinoma *in situ*, determined by two pathologists. Glandular involvement was defined as CIN extension into endocervical gland crypts on H&E sections (binary: present/absent). Individual margin status (endocervical, ectocervical, deep) was classified as positive (<1-mm clearance) or negative (>1-mm clearance). Multifocal margin involvement was categorized as none, single margin positive, or multiple margins positive (≥2 margins).

HPV molecular variables: Pre-LEEP high-risk HPV status was determined via HC2 within 3 months pre-LEEP (positive: RLU/CO ≥1.0, negative: <1.0). HPV genotype was determined via Linear Array for positive cases; women with multiple genotypes were assigned to most oncogenic type per hierarchy (HPV 16 > HPV 18 > other high-risk types). The primary genotype categories analyzed were HPV 16, HPV 18, and other high-risk types (31, 33, 35, 39, 45, 51, 52, 56, 58, 59, 68). Post-LEEP high-risk HPV status was tested at 6–9 months using identical HC2 methodology (positive/negative). Persistent HPV infection was defined as same high-risk genotype detected both pre-LEEP (within 3 months before) and post-LEEP (6–9 months after) via genotype-specific testing; coded as persistent versus non-persistent (different genotypes, cleared, or new infection).

Inflammatory biomarker: Neutrophil-to-lymphocyte ratio (NLR) was calculated from CBC obtained within 1 week pre-LEEP as absolute neutrophil count ÷ absolute lymphocyte count, analyzed continuously per one-unit increment. Samples with concurrent acute infection (fever, WBC >15,000/μL or clinical infectious diagnosis) were excluded to avoid confounding (*n* = 23, 1.0%).

Surveillance variables: First post-LEEP cytology was recorded as initial result at 6–9 months, classified per Bethesda 2014 (normal/NILM, ASC-US, LSIL, HSIL, or higher). Adjuvant management was categorized as observation only (surveillance without additional treatment) versus repeat excision (repeat LEEP, conization, or hysterectomy during follow-up). Immunosuppression status was defined as solid organ transplant with maintenance immunosuppression, autoimmune disease treated with chronic immunosuppressives (corticosteroids ≥20 mg prednisone-equivalent daily ≥3 months, DMARDs, or biologics), hematologic malignancy, or immunodeficiency disorders other than HIV, as HIV-seropositive individuals were excluded from this cohort per the exclusion criteria (binary: present/absent).

Outcome variables: Total follow-up duration was calculated from LEEP to last encounter, recurrence, death, or study end (months). Time to recurrence was measured from LEEP to histological CIN2+ confirmation for recurrent cases (months). Recurrence was defined as CIN2+ detected ≥6 months post-LEEP; events <6 months were classified as residual disease and excluded from primary analysis. Loss to follow-up was defined as failure to attend surveillance >18 months with unsuccessful contact attempts.

### 2.4 Statistical analysis

Continuous variables were evaluated for distributional assumptions using Shapiro–Wilk test alongside graphical inspection; because several measures were non-normally distributed, results are summarized as medians with interquartile ranges (IQRs). Categorical variables are reported as frequencies and proportions. Baseline comparisons between participants with and without recurrent CIN2+ were undertaken using Mann–Whitney *U*-test for continuous variables and *χ*² test or Fisher’s exact test for categorical variables as appropriate. Effect sizes were reported as rank-biserial correlation for continuous comparisons and Cramér’s V for categorical comparisons. All tests were two-sided, with statistical significance defined as *p* < 0.05. Sample size was prespecified for time-to-event prediction modeling. Assuming a 15% recurrence proportion and 16 candidate predictors in the final multivariable model, a minimum of 160 recurrent events was required consistent with contemporary recommendations for events-per-parameter ratios in time-to-event prediction modeling (45). The observed cohort accrued 334 CIN2+ recurrences, providing approximately 20.9 events per predictor and supporting stable multivariable estimation. On this basis, the available sample was considered sufficient to detect moderate associations compatible with clinical prediction modeling within a single-center cohort.

Time-to-event analyses used Cox proportional hazards regression with recurrent CIN2+ as the event. Time zero was the date of index LEEP, and participants were censored at the last documented follow-up date. Ties were handled using the Efron method. The proportional hazards assumption was evaluated using tests based on Schoenfeld residuals and inspection of log–log survival plots; no material departures were carried forward into the primary modeling strategy. Univariable Cox models were fitted for each candidate predictor to quantify crude associations, reporting hazard ratios (HRs) with 95% confidence intervals (CIs). Continuous predictors were parameterized on clinically interpretable scales to enhance interpretability and comparability: age per 10 years, body mass index per 5 kg/m², excision depth per 5 mm, and neutrophil-to-lymphocyte ratio (NLR) per one-unit increment. Predictors meeting a liberal screening threshold (*p* < 0.10) were prioritized for multivariable modeling, while clinically essential variables were retained irrespective of univariable significance to preserve face validity and alignment with established post-treatment risk frameworks.

Multivariable modeling followed a prespecified hierarchical strategy reflecting the clinical sequence of information acquisition and the conceptual pathway from host factors to lesion biology and viral persistence. Model 1 included demographics and lifestyle characteristics (age, body mass index, menopausal status, parity, smoking status, and HPV vaccination status). Model 2 added pre-LEEP disease and clinical history measures (previous cervical treatment, pre-LEEP biopsy grade, and transformation zone type). Model 3 incorporated index LEEP pathology and surgical-pathologic variables (cone depth, highest CIN grade in the specimen, glandular involvement, and multifocal margin involvement operationalized as none, single-margin positive, or multiple-margins positive). Model 4, the final nomogram model, further included HPV-related molecular variables and inflammatory biomarker information (HPV genotype grouping, persistent HPV infection defined by genotype-concordant persistence, and NLR). Incremental predictive contribution across models was assessed using likelihood ratio *χ*² tests and the Akaike information criterion (AIC). Multicollinearity was evaluated using variance inflation factors, with values >5 considered potentially problematic; all predictors retained in the final model remained below this threshold. Adjusted hazard ratios (aHRs) with 95% CIs were reported for multivariable models, and *p* < 0.05 denoted statistical significance in the final model.

Effect heterogeneity for persistent HPV infection—the dominant biological predictor—was explored across prespecified strata (age <40 vs. ≥40 years, menopausal status, highest CIN grade, margin status, vaccination status, and smoking status). Stratified Cox models were fitted within each subgroup, and multiplicative interaction terms were tested using likelihood ratio tests; non-significant interactions were interpreted as evidence of broadly consistent effects. A clinical nomogram was derived from the final multivariable Cox model by translating regression coefficients to a point-based system proportional to the β coefficients. Total points were mapped to predicted recurrence probabilities at 24 and 36 months using the baseline survival function estimated via the Breslow approach. Discrimination was quantified using Harrell’s concordance index (C-index) and time-dependent discrimination metrics at clinically relevant time horizons (6, 12, 24, 36, and 60 months), accounting for censoring. The incremental value of HPV-related predictors was assessed by comparing the discrimination of the full model with that of the model excluding HPV variables. Calibration was evaluated at 24 months by comparing predicted probabilities with observed event rates estimated by Kaplan–Meier methods across deciles of predicted risk, supplemented by the calibration slope derived from regressing observed outcomes on predicted log-risk. Overall calibration error was summarized using the Brier score. Clinical utility was examined using decision curve analysis across clinically plausible threshold probabilities for intensified surveillance, demonstrating a net benefit of nomogram-guided strategies over default “treat-all” or “treat-none” approaches across relevant risk ranges. For clinical risk stratification, the nomogram score was categorized into three pragmatic groups (low, moderate, and high) using prespecified point ranges intended to reflect actionability. Kaplan–Meier curves were constructed for these strata and compared using log-rank test, and stratum-specific cumulative recurrence estimates were reported with 95% CIs. Internal validation used bootstrap resampling (500 iterations) to estimate optimism-corrected performance, including C-index and calibration slope. Missingness was described and assessed for plausibility of missing completely at random using Little’s test. The primary analysis used complete-case modeling for variables with minimal missingness; for predictors with ≥5% missingness, sensitivity analyses used multiple imputation by chained equations (50 imputations; predictive mean matching for continuous variables and logistic or multinomial models for categorical variables), with coefficients pooled using Rubin’s rules. Additional sensitivity analyses examined robustness to alternate endpoint definitions (CIN3+ only; inclusion of early residual disease), competing-risk specification (Fine–Gray sub-distribution hazards with death as a competing event), and temporal stability by comparing model performance across calendar periods within the study window. All analyses were conducted using R (version 4.3.1) with established packages for survival modeling, prediction, calibration, decision curve analysis, and multiple imputation; confidence intervals were two-sided at the 95% level, and univariable analyses were treated as exploratory while inference focused on the prespecified final multivariable model.

## 3 Results

### 3.1 Cohort characteristics and recurrence outcomes

**Figure 1:**
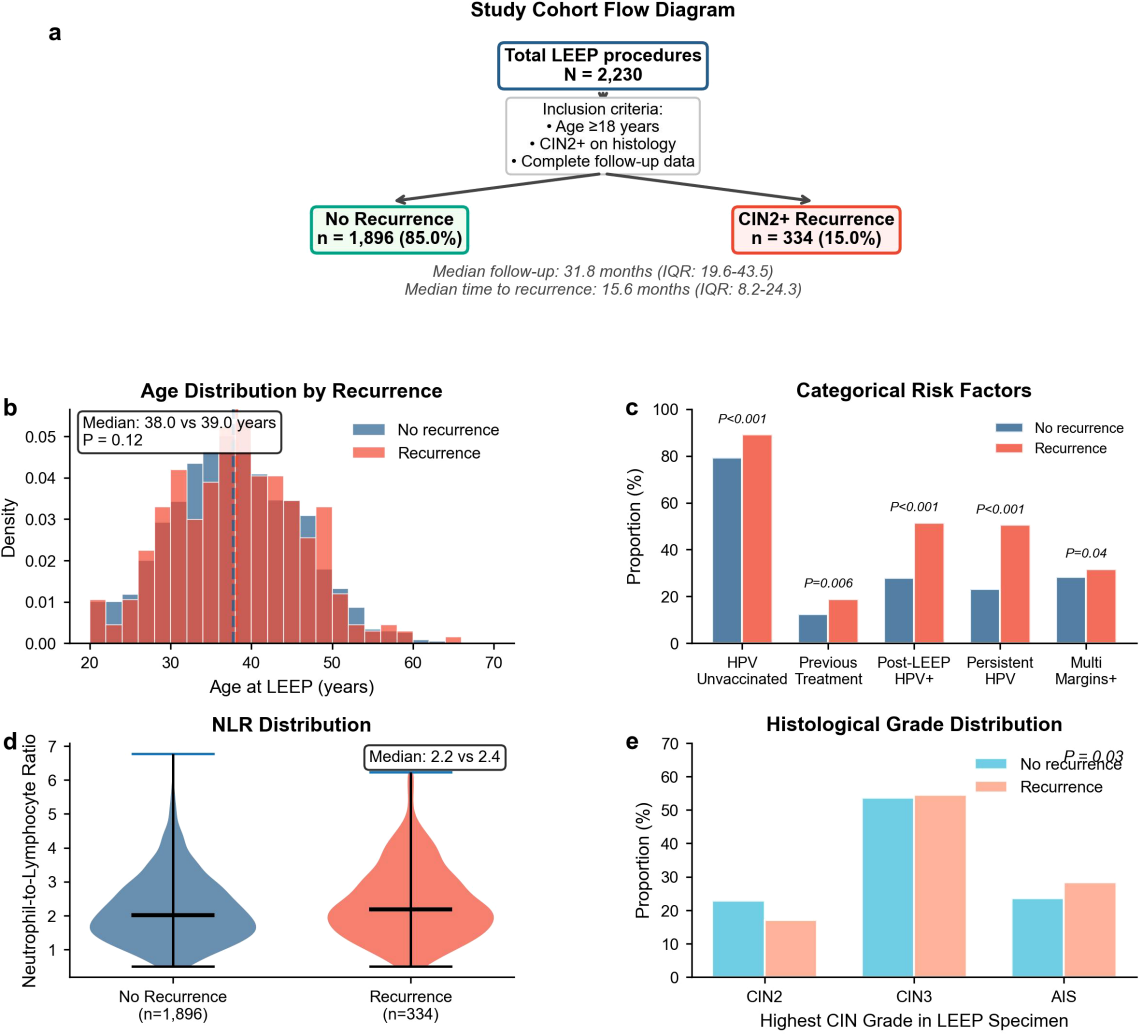
Cohort profile and baseline distributions by recurrence status. **(A)** Study flow and time-to-event summary for CIN2+ recurrence following LEEP, including follow-up duration and time-to-recurrence distribution. **(B)** Distribution of age and body mass index at index LEEP stratified by recurrence status. **(C)** Comparison of key categorical exposures by outcome, including HPV vaccination status, post-LEEP high-risk HPV status at first follow-up, persistent HPV infection (genotype concordance pre-/post-LEEP), and margin involvement category. **(D)** Distribution of pre-LEEP neutrophil-to-lymphocyte ratio (NLR) by recurrence status. **(E)** Distribution of index histopathology categories (including AIS) stratified by recurrence status. Group comparisons use Mann–Whitney *U*-tests for continuous variables and *χ*²/Fisher exact tests for categorical variables as appropriate.

A total of 2,230 women who underwent loop electrosurgical excision for CIN2+ were included, among whom 334 developed histologically confirmed CIN2+ recurrence (15.0%) (**Figure 1A**). The median follow-up duration was 31.8 months (IQR 19.6–43.5), and the median time to recurrence was 15.6 months (IQR 8.2–24.3) (**Figure 1A**). Baseline characteristics stratified by recurrence status are summarized in **Table 1**. Women who developed recurrence were more frequently postmenopausal (17.1% vs. 13.4%; *p* = 0.04) and multiparous (≥3 children: 37.4% vs. 28.6%; *p* = 0.008) and had a higher prevalence of current or former smoking (12.6% vs. 9.0%; *p* = 0.02) (**Table 1**). HPV vaccination coverage differed substantially between groups (*p* < 0.001), with a higher proportion unvaccinated among women with recurrence (89.2% vs. 79.2%) and a lower proportion fully vaccinated (2.7% vs. 7.8%) (**Table 1**; **Figure 1C**). Prior cervical treatment was more common among women with recurrence (18.9% vs. 12.5%; *p* = 0.006) (**Table 1**). In contrast, age at LEEP (median 39.0 vs. 38.0 years; *p* = 0.12) and body mass index (median 23.8 vs. 23.6 kg/m²; *p* = 0.31) did not differ materially (**Table 1**; **Figure 1B**).

**Table 1:** Baseline demographic, clinical, and pathologic characteristics of the study cohort stratified by CIN2+ recurrence status.

Characteristic	No recurrence	Recurrence	*p*-value^a^	Effect size^b^
	(*n* = 1,896)	(*n* = 334)		
Demographic and anthropometric characteristics				
Age at LEEP, median (IQR), years	38.0 (32.0–44.0)	39.0 (33.0–45.0)	0.12	0.08
Body mass index, median (IQR), kg/m²	23.6 (21.4–25.9)	23.8 (21.6–26.2)	0.31	0.05
Menopausal status, number (%)			0.04	0.12
Premenopausal	1,642 (86.6)	277 (82.9)		
Postmenopausal	254 (13.4)	57 (17.1)		
Reproductive and obstetric history				
Parity, number (%)			0.008	0.13
Nulliparous	521 (27.5)	88 (26.3)		
1 to 2 children	832 (43.9)	121 (36.2)		
≥3 children	543 (28.6)	125 (37.4)		
New sexual partner during follow-up, number (%)			0.18	0.07
No	1,754 (92.5)	302 (90.4)		
Yes	142 (7.5)	32 (9.6)		
Lifestyle and preventive factors				
Smoking status, number (%)			0.02	0.14
Never smoker	1,725 (91.0)	292 (87.4)		
Former smoker	115 (6.1)	28 (8.4)		
Current smoker	56 (3.0)	14 (4.2)		
HPV vaccination status, number (%)			<0.001	0.18
Unvaccinated	1,502 (79.2)	298 (89.2)		
Partially vaccinated	246 (13.0)	27 (8.1)		
Fully vaccinated	148 (7.8)	9 (2.7)		
Pre-LEEP disease characteristics				
Previous cervical treatment, number (%)			0.006	0.15
No	1,659 (87.5)	271 (81.1)		
Yes (cryotherapy, laser, or prior LEEP)	237 (12.5)	63 (18.9)		
Referral cytology, number (%)			0.28	0.09
ASC-US	189 (10.0)	28 (8.4)		
LSIL	378 (19.9)	62 (18.6)		
HSIL	946 (49.9)	181 (54.2)		
ASC-H	265 (14.0)	44 (13.2)		
AGC	118 (6.2)	19 (5.7)		
Pre-LEEP biopsy histology, number (%)			0.14	0.08
CIN1	284 (15.0)	41 (12.3)		
CIN2	662 (34.9)	128 (38.3)		
CIN3	950 (50.1)	165 (49.4)		
Transformation zone type, number (%)			0.09	0.1
Type 1 (fully visible SCJ)	892 (47.0)	138 (41.3)		
Type 2 (endocervical component)	743 (39.2)	144 (43.1)		
Type 3 (SCJ not fully visible)	261 (13.8)	52 (15.6)		
LEEP procedure characteristics				
Indication for LEEP, number (%)			0.51	0.03
Diagnostic	473 (24.9)	77 (23.1)		
Therapeutic	1,423 (75.1)	257 (76.9)		
Excision type, number (%)			0.42	0.04
Single-pass	1,524 (80.4)	261 (78.1)		
Multiple-pass	372 (19.6)	73 (21.9)		
Cone depth, median (IQR), mm	14.6 (11.5–17.9)	15.1 (12.1–18.3)	0.22	0.06
Index LEEP specimen pathology				
Highest CIN grade in LEEP specimen, number (%)			0.03	0.11
CIN2	433 (22.8)	57 (17.1)		
CIN3	1,016 (53.6)	182 (54.5)		
Adenocarcinoma *in situ*	447 (23.6)	95 (28.4)		
Glandular involvement, number (%)			0.08	0.09
Absent	1,374 (72.5)	226 (67.7)		
Present	522 (27.5)	108 (32.3)		
Surgical margin status				
Endocervical margin, number (%)			0.01	0.13
Negative	1,226 (64.7)	198 (59.3)		
Positive	670 (35.3)	136 (40.7)		
Ectocervical margin, number (%)			0.18	0.07
Negative	1,371 (72.3)	235 (70.4)		
Positive	525 (27.7)	99 (29.6)		
Deep/stromal margin, number (%)			0.25	0.06
Negative	1,654 (87.2)	290 (86.8)		
Positive	242 (12.8)	44 (13.2)		
Multifocal margin involvement, number (%)			0.04	0.12
None (all margins negative)	521 (27.5)	68 (20.4)		
Single margin positive	837 (44.1)	160 (47.9)		
Multiple margins positive (≥2)	538 (28.4)	106 (31.7)		
HPV status and molecular characteristics				
Pre-LEEP high-risk HPV status, number (%)			0.31	0.05
Negative	133 (7.0)	19 (5.7)		
Positive	1,763 (93.0)	315 (94.3)		
HPV genotype (if positive), number (%)			0.15	0.08
HPV 16	897 (47.3)	162 (48.5)		
HPV 18	323 (17.0)	65 (19.5)		
Other high-risk types	676 (35.7)	107 (32.0)		
Post-LEEP high-risk HPV status (first follow-up), number (%)			<0.001	0.21
Negative	1,369 (72.2)	162 (48.5)		
Positive	527 (27.8)	172 (51.5)		
Persistent HPV infection (same genotype), Number (%)			<0.001	0.32
No	1,458 (76.9)	165 (49.4)		
Yes	438 (23.1)	169 (50.6)		
Inflammatory biomarker				
Pre-LEEP neutrophil-to-lymphocyte ratio, median (IQR)	2.2 (1.8–2.7)	2.4 (1.9–2.9)	0.03	0.11
Post-LEEP surveillance and management				
First post-LEEP cytology result, number (%)			<0.001	0.24
Normal/negative	1,432 (75.5)	178 (53.3)		
ASC-US	284 (15.0)	76 (22.8)		
LSIL	123 (6.5)	48 (14.4)		
HSIL or higher	57 (3.0)	32 (9.6)		
Adjuvant management after LEEP, number (%)			0.04	0.11
Observation only	1,782 (94.0)	302 (90.4)		
Repeat excision	114 (6.0)	32 (9.6)		
Immunosuppression status, number (%)			0.62	0.03
None	1,841 (97.1)	323 (96.7)		
Present^c^	55 (2.9)	11 (3.3)		
Follow-up duration				
Total follow-up duration, median (IQR), months	32.1 (20.1–43.8)	30.2 (17.8–42.1)	0.09	0.08
Time to recurrence (for recurrent cases), median (IQR), months	—	15.6 (8.2–24.3)		

AGC, atypical glandular cells; AIS, adenocarcinoma *in situ*; ASC-H, atypical squamous cells, cannot exclude HSIL; ASC-US, atypical squamous cells of undetermined significance; CIN, cervical intraepithelial neoplasia; HPV, human papillomavirus; HSIL, high-grade squamous intraepithelial lesion; IQR, interquartile range; LEEP, loop electrosurgical excision procedure; LSIL, low-grade squamous intraepithelial lesion; SCJ, squamocolumnar junction.

^a^Continuous variables were compared using Mann–Whitney *U*-test (nonparametric test for non-normally distributed data) and are presented as median (IQR). Categorical variables were compared using the *χ*² test or Fisher exact test (when expected cell counts <5) and are presented as number (%). *p*-values <0.05 are considered statistically significant.

Effect sizes: rank-biserial correlation for continuous variables (range: 0 to 1, with 0 indicating no effect and 1 indicating perfect separation) and Cramér’s V for categorical variables (range: 0 to 1, with 0.1 = small effect, 0.3 = medium effect, 0.5 = large effect).

^c^Present immunosuppression defined as solid organ transplant with maintenance therapy, autoimmune disease on chronic immunosuppressives (corticosteroids ≥20 mg prednisone-equivalent daily ≥3 months, DMARDs, or biologics), hematologic malignancy, or immunodeficiency disorders (HIV-seropositive patients were excluded per study criteria).

With respect to index LEEP pathology, the distribution of the highest CIN grade differed between groups (*p* = 0.03), including a higher proportion of adenocarcinoma *in situ* among women with recurrence (28.4% vs. 23.6%) (**Table 1**; **Figure 1E**). Endocervical margin positivity was more frequent among women with recurrence (40.7% vs. 35.3%; *p* = 0.01) (**Table 1**). Multifocal margin involvement also differed (*p* = 0.04), with multiple positive margins observed in 31.7% of women with recurrence versus 28.4% without recurrence (**Table 1**; **Figure 1C**).

Several procedural and pathological characteristics were broadly comparable between groups, supporting the specificity of associations observed for margin burden and virological factors. Transformation zone type showed modest distributional differences that did not reach conventional statistical significance (*p* = 0.09), with type 1 observed in 41.3% versus 47.0%, type 2 in 43.1% versus 39.2%, and type 3 in 15.6% versus 13.8% among women with and without recurrence, respectively (**Table 1**). Excision technique was similar (single-pass: 78.1% vs. 80.4%; *p* = 0.42), and cone depth did not differ meaningfully (median 15.1 mm [IQR 12.1–18.3] vs. 14.6 mm [11.5–17.9]; *p* = 0.22) (**Table 1**). Glandular involvement was more frequent in the recurrence group (32.3% vs. 27.5%), although the difference was not statistically significant (*p* = 0.08), and neither ectocervical margin positivity (29.6% vs. 27.7%; *p* = 0.18) nor deep/stromal margin positivity (13.2% vs. 12.8%; *p* = 0.25) differed materially (**Table 1**).

HPV-related measures exhibited the largest between-group separations. Pre-LEEP high-risk HPV positivity was high in both groups (94.3% vs. 93.0%; *p* = 0.31), whereas post-LEEP high-risk HPV positivity at first follow-up was markedly more common among women with recurrence (51.5% vs. 27.8%; *p* < 0.001) (**Table 1**; **Figure 1C**). Persistent HPV infection (same genotype pre-/post-LEEP) occurred in 50.6% of women with recurrence compared with 23.1% without recurrence (*p* < 0.001) (**Table 1**; **Figure 1C**). HPV genotype distribution among HPV-positive women was similar between groups (*p* = 0.15), with HPV 16 detected in 48.5% versus 47.3% and HPV 18 in 19.5% versus 17.0% among women with and without recurrence, respectively (**Table 1**). Inflammatory status also differed, with a higher pre-LEEP neutrophil-to-lymphocyte ratio in the recurrence group (median 2.4 vs. 2.2; *p* = 0.03) (**Table 1**; **Figure 1D**). First post-LEEP cytology differed strongly by outcome (*p* < 0.001), including higher frequencies of ASC-US (22.8% vs. 15.0%), LSIL (14.4% vs. 6.5%), and HSIL or higher (9.6% vs. 3.0%) among women with recurrence (**Table 1**).

### 3.2 Univariable and multivariable predictors of recurrence

**Figure 2:**
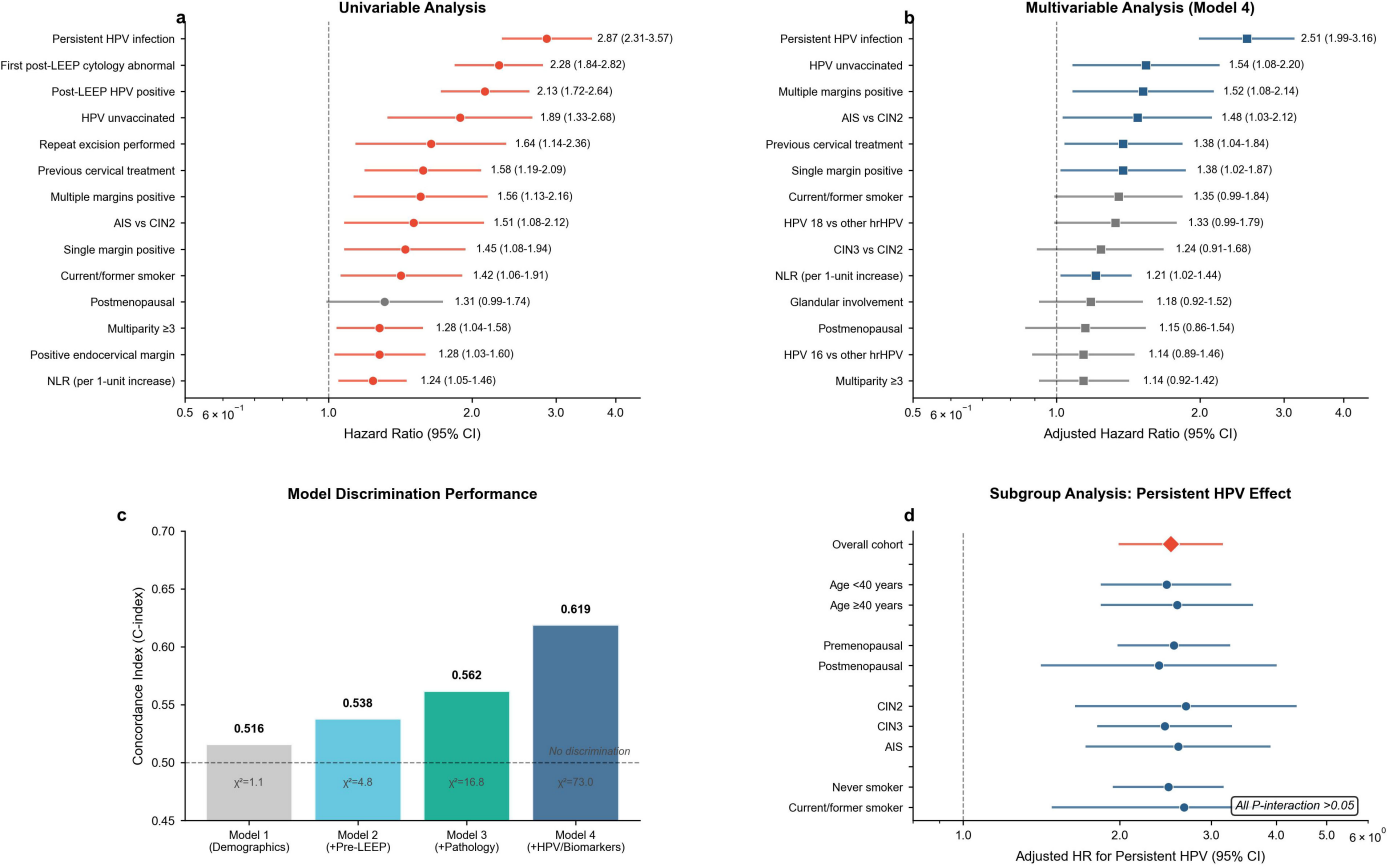
Cox regression results and incremental predictive value of hierarchical models. **(A)** Forest plot of univariable Cox proportional hazards associations with CIN2+ recurrence for candidate predictors. **(B)** Forest plot of the final multivariable Cox model used to construct the nomogram, reporting adjusted hazard ratios with 95% confidence intervals. **(C)** Model discrimination across hierarchical models (models 1–4), demonstrating incremental improvement in concordance after adding pre-LEEP characteristics, pathology, and HPV/biomarker domains. **(D)** Subgroup analysis for persistent HPV infection, presenting stratum-specific adjusted hazard ratios and tests for interaction across clinically relevant subgroups.

In univariable Cox regression (**Table 2**; **Figure 2A**), the strongest association was observed for persistent HPV infection (HR 2.87, 95% CI 2.31–3.57; *p* < 0.001). Additional predictors included abnormal first post-LEEP cytology (HR 2.28, 95% CI 1.84–2.82; *p* < 0.001), post-LEEP HPV positivity (HR 2.13, 95% CI 1.72–2.64; *p* < 0.001), and HPV unvaccinated status (HR 1.89, 95% CI 1.33–2.68; *p* < 0.001) (**Table 2**). Procedural and clinical history variables were also associated, including repeat excision (HR 1.64, 95% CI 1.14–2.36; *p* = 0.008) and previous cervical treatment (HR 1.58, 95% CI 1.19–2.09; *p* = 0.002) (**Table 2**). Pathology and margin-related predictors included multiple margins positive versus all margins negative (HR 1.56, 95% CI 1.13–2.16; *p* = 0.007), single margin positive versus all margins negative (HR 1.45, 95% CI 1.08–1.94; *p* = 0.01), and highest CIN grade AIS versus CIN2 (HR 1.51, 95% CI 1.08–2.12; *p* = 0.02) (**Table 2**). The neutrophil-to-lymphocyte ratio was also associated with recurrence risk (per one-unit increase: HR 1.24, 95% CI 1.05–1.46; *p* = 0.01) (**Table 2**). In contrast, several clinically relevant features showed weaker or non-significant univariable associations, including transformation zone type 3 versus type 1/2 (HR 1.21, 95% CI 0.90–1.64; *p* = 0.21), cone depth per 5-mm increase (HR 0.92, 95% CI 0.78–1.09; *p* = 0.33), and HPV 16/HPV 18 genotypes versus other high-risk types (**Table 2**).

**Table 2:** Univariable Cox proportional hazards analysis for time to CIN2+ recurrence.

Variable	Unadjusted hazard ratio	*p*-value	Concordance index
	(95% CI)		
Demographic and lifestyle factors			
Age (per 10-year increase)	1.03 (0.89–1.19)	0.68	0.512
Body mass index (per five-unit increase)	1.01 (0.82–1.24)	0.94	0.506
Postmenopausal (vs. premenopausal)	1.31 (0.99–1.74)	0.06	0.521
Multiparity ≥3 children (vs. 0–2)	1.28 (1.04–1.58)	0.02	0.524
Current or former smoker (vs. never)	1.42 (1.06–1.91)	0.02	0.528
Unvaccinated for HPV (vs. any vaccination)	1.89 (1.33–2.68)	<0.001	0.543
New sexual partner during follow-up (vs. none)	1.31 (0.91–1.89)	0.15	0.514
Pre-LEEP disease characteristics			
Previous cervical treatment (vs. none)	1.58 (1.19–2.09)	0.002	0.534
Referral cytology HSIL or higher (vs. ASC-US/LSIL)	1.16 (0.93–1.44)	0.19	0.518
Pre-LEEP biopsy CIN3 (vs. CIN1/CIN2)	1.09 (0.88–1.35)	0.42	0.509
Transformation zone type 3 (vs. type 1/2)	1.21 (0.90–1.64)	0.21	0.516
LEEP procedure characteristics			
Therapeutic indication (vs. diagnostic)	1.08 (0.84–1.39)	0.54	0.507
Multiple-pass excision (vs. single-pass)	1.14 (0.88–1.48)	0.32	0.513
Cone depth (per 5-mm increase)	0.92 (0.78–1.09)	0.33	0.508
Index LEEP specimen pathology			
Highest CIN grade: CIN3 vs. CIN2	1.28 (0.95–1.72)	0.11	0.516
Highest CIN grade: AIS vs. CIN2	1.51 (1.08–2.12)	0.02	0.524
Glandular involvement present (vs. absent)	1.24 (0.98–1.57)	0.07	0.518
Surgical margin status			
Positive endocervical margin (vs. negative)	1.28 (1.03–1.60)	0.03	0.526
Positive ectocervical margin (vs. negative)	1.10 (0.87–1.40)	0.42	0.509
Positive deep margin (vs. negative)	1.05 (0.76–1.45)	0.77	0.503
Multifocal margin involvement:			0.531
Single margin positive (vs. all negative)	1.45 (1.08–1.94)	0.01	
Multiple margins positive (vs. all negative)	1.56 (1.13–2.16)	0.007	
HPV status and biomarkers			
Pre-LEEP HPV positive (vs. negative)	1.27 (0.79–2.04)	0.32	0.511
HPV 16 genotype (vs. other high-risk types)	1.14 (0.92–1.42)	0.24	0.514
HPV 18 genotype (vs. other high-risk types)	1.23 (0.94–1.61)	0.13	0.517
Post-LEEP HPV positive (vs. negative)	2.13 (1.72–2.64)	<0.001	0.608
Persistent HPV infection (vs. no persistence)	2.87 (2.31–3.57)	<0.001	0.634
Neutrophil-to-lymphocyte ratio (per 1-unit increase)	1.24 (1.05–1.46)	0.01	0.529
Post-LEEP surveillance findings			
First post-LEEP cytology abnormal (vs. normal)	2.28 (1.84–2.82)	<0.001	0.615
Repeat excision performed (vs. observation only)	1.64 (1.14–2.36)	0.008	0.532
Immunosuppression present (vs. absent)	1.14 (0.62–2.09)	0.67	0.504

Each variable was analyzed separately using Cox proportional hazards regression with time to histologically confirmed CIN2+ recurrence as the outcome. Hazard ratios >1 indicate increased risk of recurrence. The concordance index (C-index) represents the discriminative ability of each individual predictor (0.5 = no discrimination; 1.0 = perfect discrimination). All models met the proportional hazards assumption based on Schoenfeld residual tests (all *p* > 0.05). *p*-values <0.05 are considered statistically significant. Variables with *p* < 0.10 in univariable analysis were considered for inclusion in multivariable models.

AIS, adenocarcinoma *in situ*; ASC-US, atypical squamous cells of undetermined significance; CI, confidence interval; CIN, cervical intraepithelial neoplasia; HPV, human papillomavirus; HSIL, high-grade squamous intraepithelial lesion; LEEP, loop electrosurgical excision procedure; LSIL, low-grade squamous intraepithelial lesion.

Progressive multivariable modeling demonstrated incremental improvement in discrimination across domains (**Figure 2C**), with the C-index increasing from 0.516 (model 1) to 0.538 (model 2), 0.562 (model 3), and 0.619 (model 4). The overall model fit for the final model was significant (likelihood ratio *χ*² = 73.0; df = 16; *p* < 0.001) (**Table 3**). In the fully adjusted model 4 (**Table 3**; **Figure 2B**), independent predictors were persistent HPV infection (aHR 2.51, 95% CI 1.99–3.16), HPV unvaccinated status (aHR 1.54, 95% CI 1.08–2.20), multiple margins positive (aHR 1.52, 95% CI 1.08–2.14), AIS versus CIN2 (aHR 1.48, 95% CI 1.03–2.12), previous cervical treatment (aHR 1.38, 95% CI 1.04–1.84), single margin positive (aHR 1.38, 95% CI 1.02–1.87), and neutrophil-to-lymphocyte ratio (per one-unit increase: aHR 1.21, 95% CI 1.02–1.44) (**Table 3**). In contrast, several covariates associated in univariable analyses did not retain independent statistical significance after full adjustment, including multiparity (aHR 1.14, 95% CI 0.92–1.42), postmenopausal status (aHR 1.15, 95% CI 0.86–1.54), and current/former smoking (aHR 1.35, 95% CI 0.99–1.84), as well as HPV 18 genotype (aHR 1.33, 95% CI 0.99–1.79) and HPV 16 genotype (aHR 1.14, 95% CI 0.89–1.46) (**Table 3**).

**Table 3:** Progressive multivariable Cox proportional hazards models with incremental adjustment for CIN2+ recurrence.

Variable	Model 1^a^	Model 2^b^	Model 3^c^	Model 4^d^
	aHR (95% CI)	aHR (95% CI)	aHR (95% CI)	aHR (95% CI)
Demographics and lifestyle				
Age (per 10-year increase)	1.01 (0.87–1.17)	1.03 (0.89–1.19)	1.06 (0.91–1.23)	1.08 (0.92–1.26)
BMI (per five-unit increase)	0.99 (0.80–1.22)	0.98 (0.79–1.21)	0.96 (0.78–1.19)	0.94 (0.76–1.16)
Postmenopausal (vs. premenopausal)	1.26 (0.95–1.68)	1.24 (0.93–1.66)	1.19 (0.89–1.60)	1.15 (0.86–1.54)
Multiparity ≥3 (vs. 0–2 children)	1.24 (1.00–1.54)	1.22 (0.98–1.52)	1.18 (0.95–1.47)	1.14 (0.92–1.42)
Current/former smoker (vs. never)	1.38 (1.02–1.86)	1.36 (1.01–1.84)	1.37 (1.01–1.85)	1.35 (0.99–1.84)
Unvaccinated for HPV (vs. vaccinated)	1.75 (1.23–2.50)	1.72 (1.20–2.45)	1.68 (1.18–2.40)	1.54 (1.08–2.20)
Pre-LEEP characteristics				
Previous cervical treatment (vs. none)	—	1.48 (1.11–1.96)	1.42 (1.07–1.89)	1.38 (1.04–1.84)
Pre-LEEP biopsy CIN3 (vs. CIN1/2)	—	1.08 (0.87–1.34)	0.98 (0.78–1.23)	0.94 (0.75–1.18)
TZ type 3 (vs. type 1/2)	—	1.16 (0.86–1.57)	1.12 (0.83–1.51)	1.09 (0.81–1.48)
LEEP pathology				
Cone depth (per 5-mm increase)	—	—	0.94 (0.79–1.11)	0.92 (0.78–1.09)
Highest CIN grade in LEEP				
CIN3 (vs. CIN2)	—	—	1.22 (0.90–1.66)	1.24 (0.91–1.68)
AIS (vs. CIN2)	—	—	1.46 (1.02–2.09)	1.48 (1.03–2.12)
Glandular involvement present (vs. absent)	—	—	1.16 (0.90–1.49)	1.18 (0.92–1.52)
Multifocal margin involvement:				
Single margin+ (vs. all negative)	—	—	1.36 (1.01–1.83)	1.38 (1.02–1.87)
Multiple margins+ (vs. all negative)	—	—	1.48 (1.06–2.08)	1.52 (1.08–2.14)
HPV and biomarkers				
HPV genotype:				
HPV 16 (vs. other hrHPV)	—	—	—	1.14 (0.89–1.46)
HPV 18 (vs. other hrHPV)	—	—	—	1.33 (0.99–1.79)
Persistent HPV infection (vs. no)	—	—	—	2.51 (1.99–3.16)
NLR (per one-unit increase)	—	—	—	1.21 (1.02–1.44)
Model fit statistics				
Concordance index (C-index)	0.516	0.538	0.562	0.619
Likelihood ratio *χ*² (df)	1.1(6)	4.8(9)	16.8(13)	73.0(16)
*p*-value for overall model	0.98	0.85	0.21	<0.001
No. of patients	2,230	2,230	2,230	2,230
No. of events	334	334	334	334

aHR, adjusted hazard ratio; AIS, adenocarcinoma *in situ*; BMI, body mass index; CI, confidence interval; CIN, cervical intraepithelial neoplasia; HPV, human papillomavirus; hrHPV, high-risk HPV; LEEP, loop electrosurgical excision procedure; NLR, neutrophil-to-lymphocyte ratio; TZ, transformation zone.

a Model 1: Demographics and lifestyle factors only (age, BMI, menopausal status, parity, smoking, HPV vaccination).

b Model 2: Model 1 + pre-LEEP characteristics (previous treatment, pre-LEEP biopsy grade, transformation zone type).

c Model 3: Model 2 + LEEP pathology (cone depth, highest CIN grade, glandular involvement, multifocal margin involvement).

d Model 4 (full): Model 3 + HPV status and biomarkers (HPV genotype, persistent infection, NLR). This is the final nomogram model. All models used Cox proportional hazards regression with time to CIN2+ recurrence as the outcome. Adjusted hazard ratios >1 indicate an increased risk of recurrence after controlling for other variables in the model. p-values <0.05 are considered statistically significant. The C-index improved from 0.516 in model 1 to 0.619 in the full model (model 4), indicating that the addition of pathology, HPV, and biomarker variables substantially improved discrimination. Model 4 formed the basis for the clinical prediction nomogram.

Subgroup analyses indicated that the association between persistent HPV infection and recurrence was consistent across clinically relevant strata (**Table 4**; **Figure 2D**). The adjusted hazard ratio was similar in women aged <40 years (2.46, 95% CI 1.84–3.28) and ≥40 years (2.58, 1.84–3.61) and remained robust across margin-burden strata (no positive margins: 2.82, 1.71–4.65; single positive: 2.41, 1.75–3.32; multiple positive: 2.48, 1.68–3.67) (**Table 4**). Effects were also comparable by vaccination status (unvaccinated: 2.56, 2.00–3.27; any vaccination: 2.21, 1.12–4.35) and smoking status (never: 2.48, 1.94–3.17; current/former: 2.66, 1.48–4.79), with no evidence of effect modification (all interaction *p*-values >0.05) (**Table 4**).

**Table 4:** Subgroup analysis: Hazard ratios for persistent HPV infection across clinically relevant subgroups.

Subgroup	No. of	No. of	Adjusted HR	*p*-value
	patients	events	(95% CI)^a^	for interaction
Age at LEEP				
<40 years	1,387	201	2.46 (1.84–3.28)	0.73
≥40 years	843	133	2.58 (1.84–3.61)	
Menopausal status				
Premenopausal	1,919	277	2.54 (1.98–3.26)	0.68
Postmenopausal	311	57	2.38 (1.41–4.01)	
Highest CIN grade in LEEP specimen				
CIN2	490	57	2.68 (1.64–4.38)	0.82
CIN3	1,198	182	2.44 (1.81–3.29)	
Adenocarcinoma in situ	542	95	2.59 (1.72–3.90)	
Multifocal margin involvement				
No positive margins	589	68	2.82 (1.71–4.65)	0.54
Single margin positive	997	160	2.41 (1.75–3.32)	
Multiple margins positive	644	106	2.48 (1.68–3.67)	
HPV vaccination status				
Unvaccinated	1,800	298	2.56 (2.00–3.27)	0.48
Any vaccination	430	36	2.21 (1.12–4.35)	
Smoking status				
Never smoker	2,017	292	2.48 (1.94–3.17)	0.79
Current or former smoker	213	42	2.66 (1.48–4.79)	
Overall cohort	2,230	334	2.51 (1.99–3.16)	—

CI, confidence interval; CIN, cervical intraepithelial neoplasia; HPV, human papillomavirus; HR, hazard ratio; LEEP, loop electrosurgical excision procedure.

a Adjusted hazard ratios for persistent HPV infection (vs. no persistence) from separate multivariable Cox models within each subgroup, adjusted for age, BMI, smoking, HPV vaccination, previous treatment, CIN grade, glandular involvement, multifocal margins, and NLR (variables from model 4, **Table 4**, excluding the stratification variable). *p*-values for interaction test whether the effect of persistent HPV infection on recurrence risk differs significantly across subgroup categories. All *p*-values >0.05 indicate that the strong association between persistent HPV and recurrence is consistent across all of the examined subgroups. The adjusted HR ranges from 2.21 to 2.82 across subgroups, demonstrating robust and clinically meaningful effect estimates regardless of patient or disease characteristics.

### 3.3 Kaplan–Meier analyses and nomogram-based risk stratification

**Figure 3:**
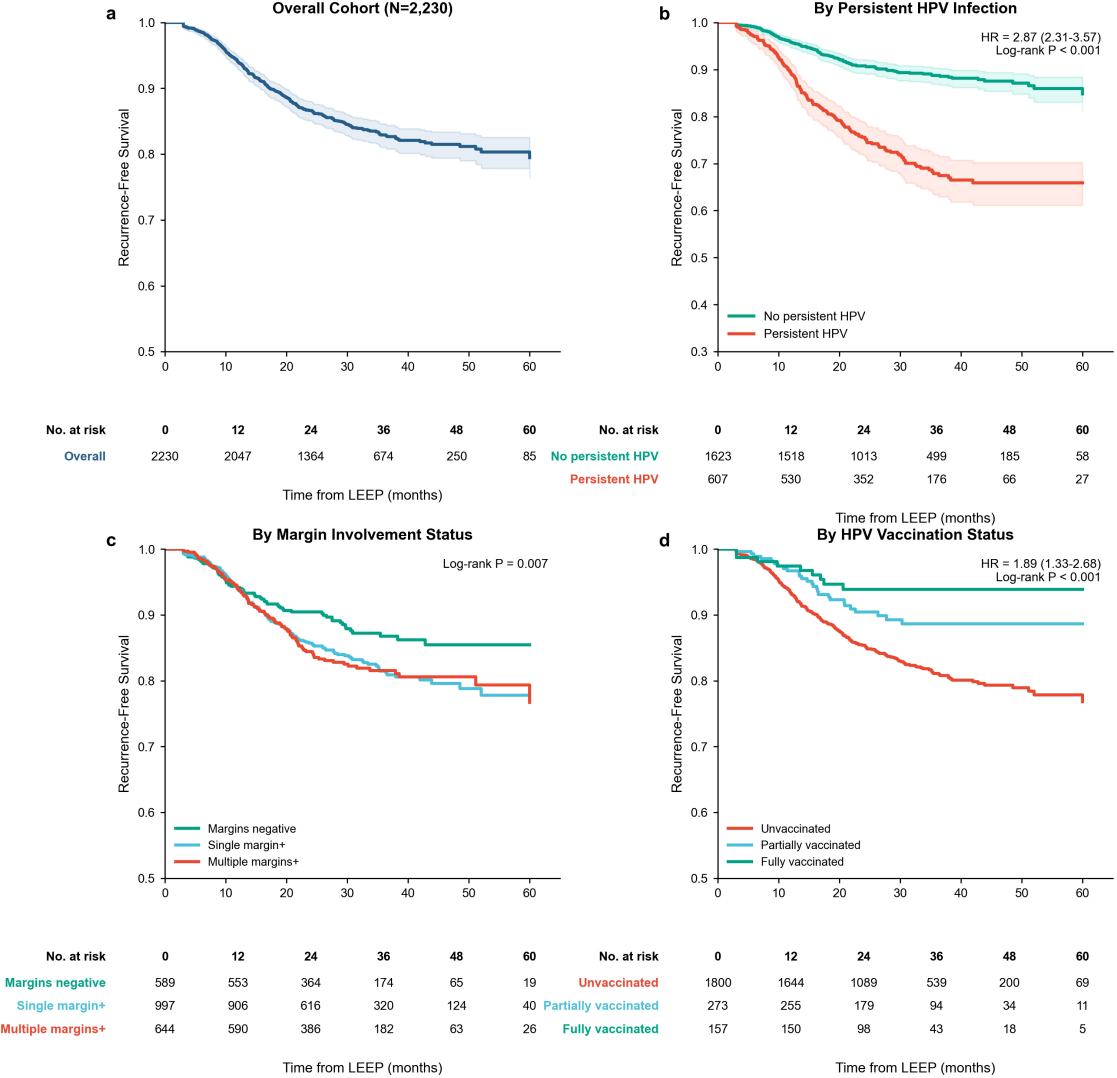
Kaplan–Meier recurrence-free survival after LEEP by key risk strata. **(A)** Overall recurrence-free survival following LEEP. **(B)** Recurrence-free survival stratified by persistent HPV infection status. **(C)** Recurrence-free survival stratified by margin involvement category (negative, single positive, multiple positive). **(D)** Recurrence-free survival stratified by HPV vaccination status (unvaccinated, partially vaccinated, fully vaccinated). *p*-values are from log-rank tests; numbers at risk are displayed beneath each panel.

**Figure 4:**
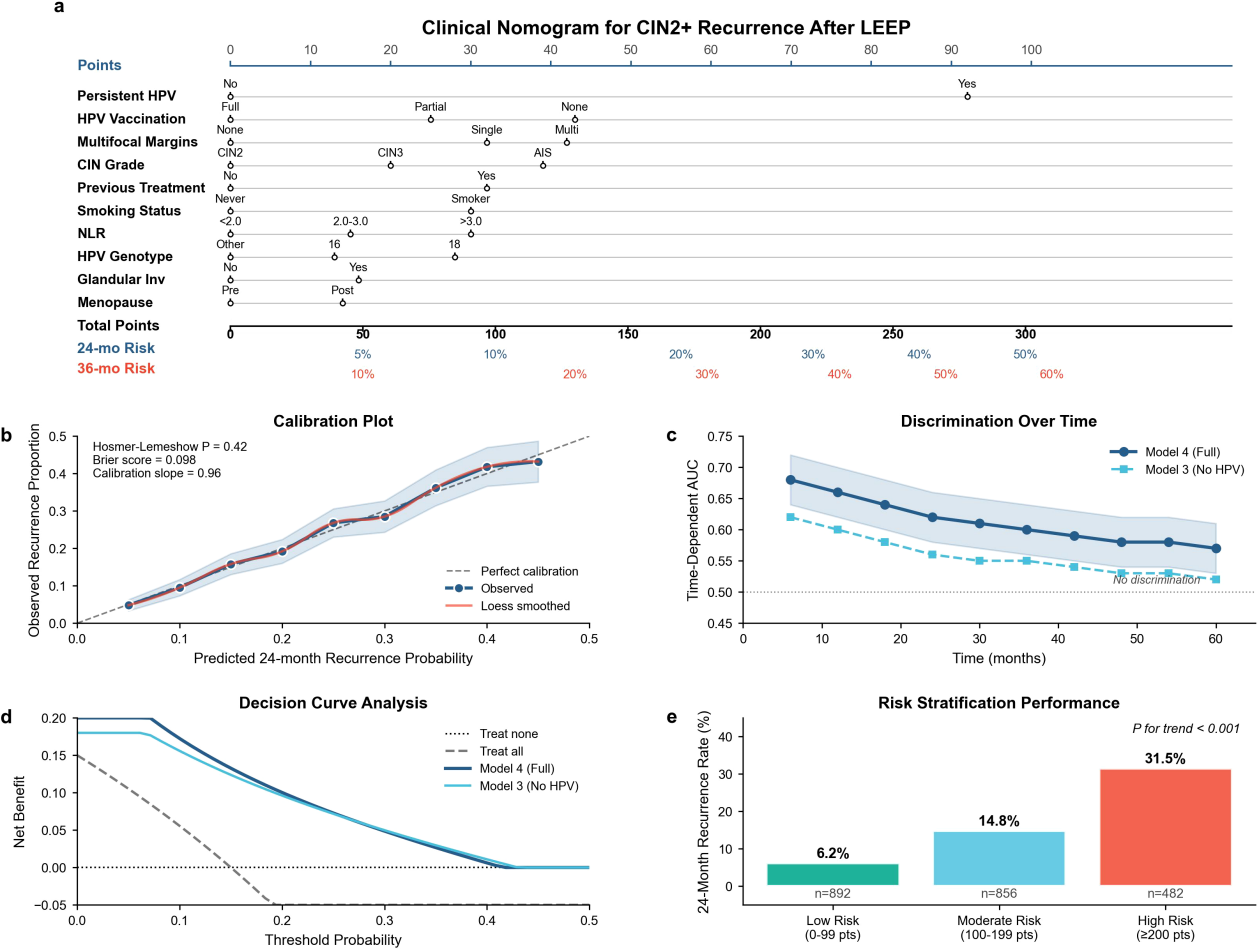
Nomogram development, calibration, and risk stratification performance. **(A)** Clinical nomogram to estimate the 24- and 36-month CIN2+ recurrence risk following LEEP based on the final multivariable model. **(B)** Calibration plot comparing predicted versus observed recurrence risk at the prespecified time horizon, with the 45° line indicating perfect calibration. **(C)** Time-dependent discrimination of the final model over follow-up. **(D)** Decision curve analysis evaluating net benefit of the nomogram across threshold probabilities compared with default strategies. **(E)** Risk group stratification (low, intermediate, high) derived from nomogram scores, with corresponding observed recurrence rates and survival separation.

Kaplan–Meier curves demonstrated pronounced separation by persistent HPV status (HR 2.87, 95% CI 2.31–3.57; log-rank *p* < 0.001) (**Figure 3B**), as well as by HPV vaccination status (HR 1.89, 95% CI 1.33–2.68; log-rank *p* < 0.001) (**Figure 3D**). Recurrence-free survival also differed by margin involvement status (log-rank *p* = 0.007) (**Figure 3C**). Numbers at risk over follow-up for each stratum are presented in **Figure 3**. A clinical nomogram incorporating the final multivariable predictors was constructed (**Figure 4A**). Calibration assessment demonstrated an acceptable agreement between predicted and observed recurrence risks, with a calibration slope of 0.96 and a Brier score of 0.098 at 24 months, indicating limited overall prediction error (**Figure 4B**). Using nomogram-derived risk stratification, patients were categorized into low-, moderate-, and high-risk groups (*n* = 892, *n* = 856, and *n* = 482, respectively), with corresponding observed 24-month recurrence rates of 6.2%, 14.8%, and 31.5% (*p* for trend <0.001) (**Figure 4E**).

## 4 Discussion

This single-center Chinese cohort study developed and internally validated a clinically interpretable nomogram for histologically confirmed CIN2+ recurrence following LEEP, integrating margin status with clinicopathological and HPV-related variables. The principal findings were that persistent post-treatment HPV infection conferred the largest independent risk (aHR 2·51), margin positivity exhibited a graded association (single and multiple positive margins both independently predictive), and absence of HPV vaccination remained independently associated with a higher recurrence risk after adjustment, underscoring the potential role of vaccination status as a clinically relevant stratification marker and a plausible target for secondary prevention counselling rather than definitive evidence of causal protection. In parallel, a modest but statistically robust association between higher pre-treatment neutrophil-to-lymphocyte ratio and recurrence was observed, while prior cervical treatment and AIS histology signaled clinically important residual or field-effect risk. Collectively, these observations support a surveillance paradigm in which virological “test-of-cure” information and excision completeness jointly drive risk stratification, with vaccination status offering an additional, actionable prevention lever.

The prominence of persistent HPV infection in the final model is consistent with mechanistic and epidemiological evidence that durable oncogenic HPV replication reflects failure of immune-mediated viral clearance and predicts ongoing neoplastic potential. Post-treatment HPV testing has repeatedly outperformed cytology alone for identifying residual or recurrent CIN, and a comprehensive meta-analysis demonstrated that HPV-based follow-up substantially improves the detection of CIN2+ after treatment compared with cytology strategies, supporting the incorporation of HPV into post-treatment care pathways (46, 47). In contrast to studies relying on single post-treatment HPV results, the present analysis emphasized genotype-concordant persistence across pre- and post-LEEP sampling, a construct that captures continued infection by the same oncogenic lineage rather than reinfection, thereby strengthening biological plausibility and clinical interpretability. Moreover, the observed magnitude of association aligns with contemporary cohorts in which persistent hrHPV, particularly HPV 16/18 persistence, dominates recurrence risk after excisional therapy, including large real-world studies and prediction-oriented analyses (12, 48, 49). These findings collectively imply that the incremental predictive gain from adding HPV persistence to pathology-only models is expected, yet the present work adds value by quantifying the extent to which HPV variables improve discrimination in a Chinese single-center setting.

Margin status remained independently associated with recurrence after accounting for HPV persistence, which is clinically salient because margins are often treated as a proxy for residual disease but can be confounded by lesion biology and operator technique (50, 51). In the current cohort, both single and multiple positive margins were retained in the final model, suggesting that the burden and distribution of involved margins provide risk information beyond a binary “positive/negative” designation. This aligns with evidence that positive margins, particularly endocervical involvement, are associated with higher post-treatment CIN2+ risk, although the strength of association varies by follow-up strategy and the incorporation of HPV testing (52). The persistence of a margin effect even after adjustment for virological status also supports a dual-pathway interpretation in which (i) incomplete excision leaves residual dysplasia that may recur early and (ii) persistent oncogenic HPV drives new or progressive lesions over time, potentially at multiple cervical sites. This distinction has practical implications: while persistent HPV may guide intensified surveillance and adjunctive preventive strategies, margin involvement may justify early re-evaluation, closer endocervical assessment, and careful consideration of repeat excision in select patients when fertility preservation and obstetric risk are balanced (53–55).

The association between HPV vaccination status and recurrence in this cohort warrants careful interpretation because post-treatment vaccination can function both as a proxy for healthcare engagement and as a biological modifier of reinfection and lesion recurrence risk (56–58). A growing evidence base supports adjuvant prophylactic HPV vaccination around the time of excisional treatment as a strategy to reduce recurrent CIN2+; a meta-analysis reported reduced recurrence among vaccinated individuals compared with unvaccinated controls, albeit with residual confounding and heterogeneity across observational designs (59–61). Notably, timing appears consequential; an updated meta-analysis focusing on vaccination relative to conization suggested that vaccination administered before or proximate to treatment may yield stronger protection than delayed vaccination, supporting the concept of immunological priming prior to potential re-exposure or residual viral replication (62). In addition, an expert consensus review has highlighted adjuvant vaccination as a plausible recurrence-prevention strategy after CIN treatment while emphasizing the need for rigorous trials and context-specific implementation planning (61, 63). Within China, where population-level coverage remains below global elimination targets and delivery is heterogeneous by region, school-based policy, and affordability, the protective signal observed here underscores the potential secondary-prevention benefits that could accrue from strengthening vaccine access for eligible women undergoing treatment (64–68). Importantly, these results should not be overextended to claim causal vaccine efficacy within this observational cohort; nonetheless, they provide locally relevant impetus for integrating vaccination counselling into post-LEEP care (69).

AIS histology emerged as an independent predictor, which is consistent with the recognized challenges of glandular disease management, including multifocality, endocervical extension, and difficulty achieving clear margins with limited excision (64, 65). Although the present cohort evaluated CIN2+ recurrence broadly, the retained AIS term likely captures a higher propensity for residual glandular lesions or synchronous multifocal disease, thereby elevating subsequent histologically confirmed events. The implication is that patients with glandular involvement may benefit from especially rigorous follow-up and, where appropriate, multidisciplinary consideration of definitive management options once reproductive plans are complete (69).

The observed association between higher neutrophil-to-lymphocyte ratio and recurrence, although modest in effect size, is biologically plausible because a systemic inflammatory response may reflect host immune competence, metabolic comorbidity, or chronic inflammatory states that impair HPV clearance (70, 71). Although NLR has been most extensively studied as a prognostic marker in invasive cervical carcinoma, its integration into a recurrence model for post-excision CIN represents an attempt to operationalize host-response heterogeneity in a clinically accessible way (72). From a translational perspective, NLR may function less as a direct causal driver than as a surrogate for immune and inflammatory milieus that modulate viral persistence; therefore, replication and mechanistic studies will be required before NLR can be recommended as a stand-alone clinical trigger (72–74).

The prediction model’s discrimination and calibration metrics indicate moderate clinical utility that is consistent with the intrinsic complexity of CIN recurrence biology (75). A C-index of 0.619 is comparable to or modestly higher than several published prediction efforts in post-conization settings, particularly those that include HPV variables and margin information, and the incremental improvement upon adding HPV measures supports their value for clinically meaningful risk stratification (12, 48, 76). The observed gradient in 24-month recurrence rates across low-, intermediate-, and high-risk groups suggests that the model can support practical pathways such as shorter-interval HPV-based follow-up for high-risk individuals and standard follow-up for low-risk individuals, thereby preserving resources while prioritizing those at greatest risk.

These findings should be interpreted within the broader Chinese cervical cancer control landscape, where scale-up of vaccination and organized screening remain central to elimination goals. Modeling analyses indicate that a high coverage of HPV vaccination combined with effective screening can drive substantial reductions in cervical cancer burden and move countries toward elimination thresholds (77–79). China-specific projections similarly suggest that optimized combinations of vaccination and screening are required to bend incidence trajectories over the coming decades, with policy decisions influencing the speed and equity of impact (80, 81). In that context, improving post-treatment management is a complementary, near-term strategy: although it does not replace primary prevention, it mitigates recurrent disease, reduces repeat procedures, and may prevent progression among women already on a high-risk pathway.

Several strengths enhance the interpretability of this study. First, the outcome definition required a histological confirmation of CIN2+, which reduces misclassification compared with cytology-only recurrence definitions. Second, the modeling strategy was clinically sequenced and integrated virological status with pathology and host-response variables, thereby reflecting real-world decision points. Third, internal validation procedures and calibration assessment support that predicted risks were not grossly overfit to the derivation data. Several limitations should be acknowledged. First, the single-center, retrospective design limits generalizability because case-mix, operator technique, pathology interpretation, follow-up intensity, and HPV assay performance may differ across Chinese regions and care tiers, thereby affecting both absolute recurrence risk and model calibration. Second, we excluded HIV-seropositive patients to maintain cohort homogeneity for this initial model development, given the well-documented effects of HIV on HPV persistence, accelerated CIN progression, and substantially elevated post-treatment recurrence risk. This exclusion limits the applicability of our nomogram to immunocompetent populations and represents an important area for future investigation. HIV status is a clinically relevant risk modifier, and future studies should include HIV-seropositive women to develop HIV-specific risk stratification tools or to evaluate whether HIV status warrants separate surveillance algorithms, as immunosuppressed populations may require intensified follow-up protocols regardless of other risk factors. Third, external validation was not performed; consequently, transportability and clinical utility in independent populations remain uncertain despite acceptable internal performance. Fourth, residual confounding is plausible, particularly for HPV vaccination status and behavioral factors (e.g., smoking) because vaccination timing relative to treatment, vaccine type, and indications for vaccination were not fully captured, and vaccinated individuals may differ systematically in health-seeking behaviors and surveillance adherence. Fifth, HPV persistence was defined using available genotyping, yet incomplete genotype panels, interval censoring, or transient clearance with later redetection could introduce misclassification; similarly, post-treatment HPV and cytology measures are susceptible to timing variability and may not be uniformly available in all settings. Sixth, several potentially important determinants were unavailable or not standardized, including lesion size/volume, colposcopy impression, detailed margin length or thermal artefact, immunosuppression and other comorbidities, and molecular markers (e.g., p16/Ki-67, viral integration), which may have improved discrimination beyond the observed moderate performance. Vaccination timing relative to excision was not uniformly available, precluding stratified analyses by pre- versus post-treatment immunization. Finally, recurrence ascertainment depends on follow-up completeness; although histological confirmation strengthens outcome validity, informative loss to follow-up could bias estimates if related to risk status.

## 5 Conclusion

This single-center Chinese cohort study developed and internally validated a clinically interpretable nomogram to predict histologically confirmed CIN2+ recurrence after LEEP, integrating margin status with clinicopathological and virological determinants. Persistent high-risk HPV infection emerged as the dominant independent predictor of recurrence, whereas margin positivity exhibited a graded association, supporting the complementary contributions of incomplete excision-related residual disease and subsequent virologically driven neoplastic recurrence to post-treatment failure. Lack of HPV vaccination remained associated with increased recurrence risk after adjustment, underscoring the potential value of adjuvant vaccination counselling and access within secondary prevention pathways. In addition, prior cervical treatment, AIS histology, and elevated neutrophil-to-lymphocyte ratio contributed incremental prognostic information, suggesting that host inflammatory milieu may modulate viral persistence and recurrence susceptibility. Overall, the model achieved moderate discrimination with acceptable calibration and clearly separated low-, intermediate-, and high-risk strata, providing a pragmatic, moderately discriminative foundation for risk-adapted surveillance and informed prioritization of follow-up intensity. External multicenter validation is warranted before broader clinical implementation. Future studies should include HIV-seropositive patients to evaluate whether HIV status warrants modified risk stratification or intensified surveillance protocols for this immunosuppressed subgroup.

## Data Availability

The raw data supporting the conclusions of this article will be made available by the authors, without undue reservation.

## References

[1] Li Y Hou X Chen W Wang S Ma X . Development and validation of a nomogram for predicting recurrence-free survival in endometrial cancer: a multicenter study. Sci Rep. (2023) 13:20270. doi: 10.1038/s41598-023-47419-8, PMID: 37985680 PMC10662280

[2] Li Z Liu P Yin A Zhang B Xu J Chen Z . Global landscape of cervical cancer incidence and mortality in 2022 and predictions to 2030: The urgent need to address inequalities in cervical cancer. Int J Cancer. (2025) 157:288–97. doi: 10.1002/ijc.35369, PMID: 40026066

[3] Das M . WHO launches strategy to accelerate elimination of cervical cancer. Lancet Oncol. (2021) 22:20–1. doi: 10.1016/s1470-2045(20)30729-4, PMID: 33248466

[4] Arbyn M Weiderpass E Bruni L de Sanjosé S Saraiya M Ferlay J . Estimates of incidence and mortality of cervical cancer in 2018: a worldwide analysis. Lancet Global Health. (2020) 8:e191–203. doi: 10.1016/s2214-109x(19)30482-6, PMID: 31812369 PMC7025157

[5] Cohen PA Jhingran A Oaknin A Denny L . Cervical cancer. Lancet (London England). (2019) 393:169–82. doi: 10.1016/s0140-6736(18)32470-x, PMID: 30638582

[6] Huang J Deng Y Boakye D Tin MS Lok V Zhang L . Global distribution, risk factors, and recent trends for cervical cancer: A worldwide country-level analysis. Gynecol Oncol. (2022) 164:85–92. doi: 10.1016/j.ygyno.2021.11.005, PMID: 34799136

[7] Wu J Jin Q Zhang Y Ji Y Li J Liu X . Global burden of cervical cancer: current estimates, temporal trend and future projections based on the GLOBOCAN 2022. J Natl Cancer Center. (2025) 5:322–9. doi: 10.1016/j.jncc.2024.11.006, PMID: 40693230 PMC12276544

[8] Santesso N Mustafa RA Wiercioch W Kehar R Gandhi S Chen Y . Systematic reviews and meta-analyses of benefits and harms of cryotherapy, LEEP, and cold knife conization to treat cervical intraepithelial neoplasia. Int J Gynaecol Obstet. (2016) 132:266–71. doi: 10.1016/j.ijgo.2015.07.026, PMID: 26643302

[9] El-Nashar SA Shazly SA Hopkins MR Bakkum-Gamez JN Famuyide AO . Loop electrosurgical excision procedure instead of cold-knife conization for cervical intraepithelial neoplasia in women with unsatisfactory colposcopic examinations: A systematic review and meta-analysis. J Lower Genit Tract Dis. (2017) 21:129–36. doi: 10.1097/lgt.0000000000000287, PMID: 27977541

[10] Khunnarong J Bunyasontikul N Tangjitgamol S . Treatment outcomes of patients with cervical intraepithelial neoplasia or invasive carcinoma who underwent loop electrosurgical excision procedure. World J Oncol. (2021) 12:111–8. doi: 10.14740/wjon1391, PMID: 34349855 PMC8297047

[11] Bradbury M Rabasa J Murcia MT Dinarès MC Sainz A Castellet C . Can we reduce overtreatment of cervical high-grade squamous intraepithelial lesions? J Lower Genit Tract Dis. (2022) 26:20–6. doi: 10.1097/lgt.0000000000000635, PMID: 34928250

[12] Ding T Li L Duan R Chen Y Yang B Xi M . Risk factors analysis of recurrent disease after treatment with a loop electrosurgical excision procedure for high-grade cervical intraepithelial neoplasia. Int J Gynaecol Obstet. (2023) 160:538–47. doi: 10.1002/ijgo.14340, PMID: 35810389 PMC10087663

[13] Shen H Wu MH Lin JJ Hsu HC Tai YJ Wu CY . Risk factors of recurrent cervical intraepithelial neoplasm 2/3 after primary excisional conization/LEEP treatments: A follow-up nationwide cohort study. Eur J Obstet Gynecol Reprod Biol: X. (2025) 26:100397. doi: 10.1016/j.eurox.2025.100397, PMID: 40491856 PMC12148459

[14] Wu D Zheng Y Chen W Guo C Yu J Chen G . Prediction of residual/recurrent disease by HPV genotype after loop excision procedure for high-grade cervical intraepithelial neoplasia with negative margins. Aust New Z J Obstet Gynaecol. (2011) 51:114–8. doi: 10.1111/j.1479-828X.2010.01280.x, PMID: 21466511

[15] Bogani G Sopracordevole F Ciavattini A Vizza E Vercellini P Giannini A . Duration of human papillomavirus persistence and its relationship with recurrent cervical dysplasia. Eur J Cancer Prev. (2023) 32:525–32. doi: 10.1097/cej.0000000000000822, PMID: 37401466

[16] Mariani L Sandri MT Preti M Origoni M Costa S Cristoforoni P . HPV-testing in follow-up of patients treated for CIN2+ Lesions. J Cancer. (2016) 7:107–14. doi: 10.7150/jca.13503, PMID: 26722366 PMC4679387

[17] Zhang J Wu L Zhu Y Liu G Wang D . Human papillomavirus infection and disease recurrence/persistence after treatment for women of high-grade cervical intraepithelial neoplasia with coexisting vaginal intraepithelial neoplasia. Front Cell Infect Microbiol. (2025) 15:1602216. doi: 10.3389/fcimb.2025.1602216, PMID: 40703673 PMC12283611

[18] Bruno MT Valenti G Ruggeri Z Incognito GG Coretti P Montana GD . Correlation of the HPV 16 genotype persistence in women undergoing LEEP for CIN3 with the risk of CIN2+ Relapses in the first 18 months of follow-up: A multicenter retrospective study. Diagn (Basel Switzerland). (2024) 14:509. doi: 10.3390/diagnostics14050509, PMID: 38472983 PMC10931513

[19] Tong Y Xu L Sun Y Lan Y Zhang K . Construction of a nomogram model for predicting residual or recurrent cervical intraepithelial neoplasia after the loop electrosurgical excision procedure. Am J Clin Pathol. (2025) 165:aqaf125. doi: 10.1093/ajcp/aqaf125, PMID: 41334789

[20] Duan R Qiao Y Clifford G Zhao F . Cancer burden attributable to human papillomavirus infection by sex, cancer site, age, and geographical area in China. Cancer Med. (2020) 9:374–84. doi: 10.1002/cam4.2697, PMID: 31714036 PMC6943148

[21] Yan H Wang Q Qiao Y . Cervical cancer prevention in China: where are we now, and what’s next? Cancer Biol Med. (2024) 21:213–7. doi: 10.20892/j.issn.2095-3941.2023.0432, PMID: 38172555 PMC10976326

[22] An J Liu Y Ma Y Jiao YZ Liang XF Jin N . Real-world data of China: Analysis of HPV vaccine coverage and post-vaccination adverse reaction monitoring in Western Chinese provinces from 2018 to 2021. Hum Vaccines Immunotherapeut. (2024) 20:2315653. doi: 10.1080/21645515.2024.2315653, PMID: 38372046 PMC10878016

[23] Ma S Sun K Han B Zheng R Wei W . Cervical cancer burden and trends in China, 2000-2020: Asia-Pacific international comparisons and insights for elimination goals. Cancer Biol Med. (2025) 22:1017–35. doi: 10.20892/j.issn.2095-3941.2025.0386, PMID: 41054968 PMC12501890

[24] Hu SY Zhao XL Zhang Y Qiao YL Zhao FH . Interpretation of “WHO guideline for screening and treatment of cervical pre-cancer lesions for cervical cancer prevention, second edition”. Zhonghua Yi Xue Za Zhi. (2021) 101:2653–7. doi: 10.3760/cma.j.cn112137-20210719-01609, PMID: 34404156

[25] Campos NG Tsu V Jeronimo J Regan C Resch S Clark A . Health impact of delayed implementation of cervical cancer screening programs in India: A modeling analysis. Int J Cancer. (2019) 144:687–96. doi: 10.1002/ijc.31823, PMID: 30132850 PMC6519250

[26] Zhang J Zha T Wang X He W . Prevalence and genotype distribution of HPV infections among women in Chengdu,China. Virol J. (2024) 21:52. doi: 10.1186/s12985-024-02317-x, PMID: 38429823 PMC10908056

[27] Qiu B Jiang N Jiang J Mao X Wang X . The prevalence and genotype distribution of high-risk human papillomaviruses among women in Xianning, China. Virol J. (2024) 21:140. doi: 10.1186/s12985-024-02413-y, PMID: 38890675 PMC11186159

[28] Pulvirenti A Pea A Chang DK Jamieson NB . Clinical and molecular risk factors for recurrence following radical surgery of well-differentiated pancreatic neuroendocrine tumors. Front Med. (2020) 7:385. doi: 10.3389/fmed.2020.00385, PMID: 32850899 PMC7419466

[29] Zhang P Li YL Qiu XD Luo J Shi YF Sun YL . Clinicopathological characteristics and risk factors for recurrence of well-differentiated pancreatic neuroendocrine tumors after radical surgery: a case-control study. World J Surg Oncol. (2019) 17:66. doi: 10.1186/s12957-019-1606-8, PMID: 30975157 PMC6460793

[30] Zhai F Mu S Song Y Zhang M Zhang C Lv Z . Machine learning prediction of residual and recurrent high-grade CIN post-LEEP. Cancer Manage Res. (2024) 16:1175–87. doi: 10.2147/cmar.s484057, PMID: 39258245 PMC11385362

[31] Deng L Wang T Chen Y Tang X Xiang D . A predictive model for residual lesions after LEEP surgery in CIN III patients. Front Med. (2023) 10:1326833. doi: 10.3389/fmed.2023.1326833, PMID: 38148909 PMC10751019

[32] Cheng Z Zhao Y Xu T Wu L Zhao H Li J . Epidemiological characteristics and genotype distribution of human papillomavirus infection in Yangpu district, Shanghai, 2020-2024. Virol J. (2025) 22:225. doi: 10.1186/s12985-025-02855-y, PMID: 40624568 PMC12232720

[33] Li Y Zhao F Wu D Qin C Lu Y Yang Y . Prevalence of human papillomavirus and genotype distribution in chinese men: A systematic review and meta-analysis. Cancer Med. (2025) 14:e70686. doi: 10.1002/cam4.70686, PMID: 39960180 PMC11831462

[34] Lin W Huang Y Zhang Y Huang L Cai H Huang G . Risk of residual/recurrent cervical diseases in HPV-positive women post-conization depends on HPV integration status. Infect Agents Cancer. (2025) 20:5. doi: 10.1186/s13027-025-00637-3, PMID: 39875925 PMC11773928

[35] Zheng H Gao M Xu H Cai J Wu Z Gu Y . Initial different human papillomavirus infection statuses and subsequent infection risk: a decade-long longitudinal study. J Health Popul Nutr. (2025) 45:32. doi: 10.1186/s41043-025-01202-9, PMID: 41454363 PMC12853600

[36] Griswold D Venturini S Carney N Rubiano AM Hutchinson PJ Kolias AG . Development, implementation and validation of resource-stratified guidelines in low-income and middle-income countries: a scoping review protocol. BMJ Open. (2022) 12:e059603. doi: 10.1136/bmjopen-2021-059603, PMID: 36171036 PMC9528583

[37] Abdi YH Abdi MS Bashir SG Ahmed NI Abdullahi YB . Understanding global health inequality and inequity: causes, consequences, and the path toward justice in healthcare. Public Health Chall. (2025) 4:e70156. doi: 10.1002/puh2.70156, PMID: 41146749 PMC12553980

[38] von Elm E Altman DG Egger M Pocock SJ Gøtzsche PC Vandenbroucke JP . The Strengthening the Reporting of Observational Studies in Epidemiology (STROBE) statement: guidelines for reporting observational studies. Lancet (London England). (2007) 370:1453–7. doi: 10.1016/s0140-6736(07)61602-x, PMID: 18064739

[39] Collins GS Reitsma JB Altman DG Moons KG . Transparent reporting of a multivariable prediction model for individual prognosis or diagnosis (TRIPOD): the TRIPOD statement. BMJ (Clinical Res ed). (2015) 350:g7594. doi: 10.1136/bmj.g7594, PMID: 25569120

[40] Nayar R Wilbur DC . The bethesda system for reporting cervical cytology: A historical perspective. Acta Cytolog. (2017) 61:359–72. doi: 10.1159/000477556, PMID: 28693017

[41] Bornstein J Bentley J Bösze P Girardi F Haefner H Menton M . 2011 colposcopic terminology of the International Federation for Cervical Pathology and Colposcopy. Obstet Gynecol. (2012) 120:166–72. doi: 10.1097/AOG.0b013e318254f90c, PMID: 22914406

[42] Perkins RB Guido RS Castle PE Chelmow D Einstein MH Garcia F . 2019 ASCCP risk-based management consensus guidelines for abnormal cervical cancer screening tests and cancer precursors. J Lower Genit Tract Dis. (2020) 24:102–31. doi: 10.1097/lgt.0000000000000525, PMID: 32243307 PMC7147428

[43] Li M Wei L Sui L Ma D Kong B Wu X . Guidelines for cervical cancer screening in China. Gynecol Obstet Clin Med. (2023) 3:189–94. doi: 10.1016/j.gocm.2023.10.005, PMID: 41743167

[44] WHO Expert Consultation . Appropriate body-mass index for Asian populations and its implications for policy and intervention strategies. Lancet (London England). (2004) 363:157–63. doi: 10.1016/s0140-6736(03)15268-3, PMID: 14726171

[45] Vittinghoff E McCulloch CE . Relaxing the rule of ten events per variable in logistic and Cox regression. Am J Epidemiol. (2007) 165:710–8. doi: 10.1093/aje/kwk052, PMID: 17182981

[46] Onuki M Matsumoto K Sakurai M Ochi H Minaguchi T Satoh T . Posttreatment human papillomavirus testing for residual or recurrent high-grade cervical intraepithelial neoplasia: a pooled analysis. J Gynecol Oncol. (2016) 27:e3. doi: 10.3802/jgo.2016.27.e3, PMID: 26463429 PMC4695453

[47] Arbyn M Paraskevaidis E Martin-Hirsch P Prendiville W Dillner J . Clinical utility of HPV-DNA detection: triage of minor cervical lesions, follow-up of women treated for high-grade CIN: an update of pooled evidence. Gynecol Oncol. (2005) 99:S7–11. doi: 10.1016/j.ygyno.2005.07.033, PMID: 16154623

[48] Lu CH Liu FS Kuo CJ Chang CC Ho ES . Prediction of persistence or recurrence after conization for cervical intraepithelial neoplasia III. Obstet Gynecol. (2006) 107:830–5. doi: 10.1097/01.AOG.0000206777.28541.fc, PMID: 16582119

[49] Bogani G Lalli L Sopracordevole F Ciavattini A Ghelardi A Simoncini T . Development of a nomogram predicting the risk of persistence/recurrence of cervical dysplasia. Vaccines. (2022) 10:579. doi: 10.3390/vaccines10040579, PMID: 35455328 PMC9029732

[50] Ausburn M Pyne JM Day AT Hajnas N Moon D Myers LL . Margin status and recurrence in surgically treated patients with HPV+ Oropharyngeal cancer. Laryngoscope. (2025) 135:2777–82. doi: 10.1002/lary.32091, PMID: 40035290

[51] Feng H Chen H Huang D He S Xue Z Pan Z . Relationship between positive margin and residual/recurrence after excision of cervical intraepithelial neoplasia: a systematic review and meta-analysis. Trans Cancer Res. (2022) 11:1762–9. doi: 10.21037/tcr-22-1466, PMID: 35836541 PMC9273651

[52] Abdulaziz AMA You X Liu L Sun Y Zhang J Sun S . Management of high-grade squamous intraepithelial lesion patients with positive margin after LEEP conization: A retrospective study. Medicine. (2021) 100:e26030. doi: 10.1097/md.0000000000026030, PMID: 34011112 PMC8137043

[53] Koshiol J Lindsay L Pimenta JM Poole C Jenkins D Smith JS . Persistent human papillomavirus infection and cervical neoplasia: a systematic review and meta-analysis. Am J Epidemiol. (2008) 168:123–37. doi: 10.1093/aje/kwn036, PMID: 18483125 PMC2878094

[54] Arbyn M Xu L Simoens C Martin-Hirsch PP . Prophylactic vaccination against human papillomaviruses to prevent cervical cancer and its precursors. Cochrane Database Syst Rev. (2018) 5:Cd009069. doi: 10.1002/14651858.CD009069.pub3, PMID: 29740819 PMC6494566

[55] Awan UA Khattak AA Bai Q Khan S . Pakistan’s transgender health disparities-a threat to HPV elimination? Lancet Reg Health Southeast Asia. (2024) 24:100351. doi: 10.1016/j.lansea.2024.100351, PMID: 38756159 PMC11096673

[56] Awan UA Guo X Khattak AA Hassan U Bashir S . HPV vaccination and cervical cancer screening in Afghanistan threatened. Lancet Infect Dis. (2023) 23:141–2. doi: 10.1016/s1473-3099(22)00868-4, PMID: 36610440

[57] Awan UA Naeem W Khattak AA Mahmood T Kamran S Khan S . An exploratory study of knowledge, attitudes, and practices toward HPV associated anal cancer among Pakistani population. Front Oncol. (2023) 13:1257401. doi: 10.3389/fonc.2023.1257401, PMID: 37954070 PMC10637352

[58] Awan UA Zafar N Khan SN Ahmed N Saadia Z Khan S . Low HPV vaccination coverage in Pakistan: when misinformation undermines infectious disease prevention. J Infect. (2026) 92:106674. doi: 10.1016/j.jinf.2025.106674, PMID: 41485493

[59] Awan UA Khattak AA Ahmed N Guo X Akhtar S Kamran S . An updated systemic review and meta-analysis on human papillomavirus in breast carcinogenesis. Front Oncol. (2023) 13:1219161. doi: 10.3389/fonc.2023.1219161, PMID: 37711194 PMC10498127

[60] Awan UA Bashir S Hassan U Khan SN Awan FM Jabbar A . HPV-driven breast carcinogenesis: associations with tumor severity, Ki67 expression and metastasis. Infect Agents Cancer. (2025) 20:55. doi: 10.1186/s13027-025-00668-w, PMID: 40804747 PMC12345114

[61] Di Donato V Caruso G Petrillo M Kontopantelis E Palaia I Perniola G . Adjuvant HPV vaccination to prevent recurrent cervical dysplasia after surgical treatment: A meta-analysis. Vaccines. (2021) 9:410. doi: 10.3390/vaccines9050410, PMID: 33919003 PMC8143003

[62] Jentschke M Kampers J Becker J Sibbertsen P Hillemanns P . Prophylactic HPV vaccination after conization: A systematic review and meta-analysis. Vaccine. (2020) 38:6402–9. doi: 10.1016/j.vaccine.2020.07.055, PMID: 32762871

[63] Han L Zhang B . Can prophylactic HPV vaccination reduce the recurrence of cervical lesions after surgery? Review and prospect. Infect Agents Cancer. (2023) 18:66. doi: 10.1186/s13027-023-00547-2, PMID: 37898754 PMC10613367

[64] Sung H Ferlay J Siegel RL Laversanne M Soerjomataram I Jemal A . Global cancer statistics 2020: GLOBOCAN estimates of incidence and mortality worldwide for 36 cancers in 185 countries. CA: Cancer J Clin. (2021) 71:209–49. doi: 10.3322/caac.21660, PMID: 33538338

[65] Awan UA Song Q Ciombor KK Toriola AT Choi J Su T . Demographic and clinicopathologic factors associated with colorectal adenoma recurrence. JAMA Netw Open. (2026) 9:e2556853. doi: 10.1001/jamanetworkopen.2025.56853, PMID: 41637071 PMC12873766

[66] Chen Y Jiang N Jiao Y Chen J Cao A An J . Analysis of HPV vaccination and influencing factors among 9-14-year-old girls in underdeveloped areas of northwestern China: A cross-sectional survey report on guardians. Vaccine. (2025) 62:127568. doi: 10.1016/j.vaccine.2025.127568, PMID: 40780093

[67] Yang L Xing C Yu X Xu Y Wang W Chang C . Coverage of HPV vaccination and influencing factors among female college students in northern China. Vaccines. (2025) 13:598. doi: 10.3390/vaccines13060598, PMID: 40573929 PMC12197517

[68] Bibi S Javed F Fozan M Shafique E Tajummal A Babar MM . Assessing cervical cancer and HPV awareness in Pakistan’s medical college students pursuing healthcare careers. BioSci Rev. (2025) 7:33–48. doi: 10.32350/bsr.74.04

[69] Aviraj KS Wasnik A Gupta L Ranjan A Suresh H . Effectiveness of interventions to improve vaccine efficacy: a systematic review and meta-analysis. Syst Rev. (2025) 14:105. doi: 10.1186/s13643-025-02856-6, PMID: 40346627 PMC12063308

[70] Sarrafan-Chaharsoughi Z Sinaii N Demidowich AP Yanovski JA . The association of Neutrophil-to-Lymphocyte ratio with metabolic syndrome in U.S. Adults: Findings from the 1999–2018 National Health and Nutrition Examination survey. J Clin Trans Endocrinol. (2025) 39:100382. doi: 10.1016/j.jcte.2024.100382, PMID: 39790833 PMC11714674

[71] Ahmad Ganaie Z Aqel Y Almaalouli B Alsarkhi LN Kabir Dar A Maali Abusal A . Association between elevated neutrophil-to-lymphocyte ratio and mortality risk in community-acquired pneumonia: A systematic review and meta-analysis. Cureus. (2025) 17:e93292. doi: 10.7759/cureus.93292, PMID: 41146762 PMC12554325

[72] Origoni M Cantatore F Candotti G Candiani M . Prognostic significance of neutrophil/lymphocytes ratio (NLR) in predicting recurrence of cervical dysplasia. BioMed Res Int. (2022) 2022:1149789. doi: 10.1155/2022/1149789, PMID: 35445135 PMC9015869

[73] Regolo M Vaccaro M Sorce A Stancanelli B Colaci M Natoli G . Neutrophil-to-lymphocyte ratio (NLR) is a promising predictor of mortality and admission to intensive care unit of COVID-19 patients. J Clin Med. (2022) 11:2235. doi: 10.3390/jcm11082235, PMID: 35456328 PMC9027549

[74] Siahaan PP Widiarti W Saputra PBT Putra RM D’Oria M . Neutrophil-to-lymphocyte ratio as a potential biomarker in predicting in-stent restenosis: A systematic review and meta-analysis. PloS One. (2025) 20:e0322461. doi: 10.1371/journal.pone.0322461, PMID: 40378151 PMC12083799

[75] Van Calster B Nieboer D Vergouwe Y De Cock B Pencina MJ Steyerberg EW . A calibration hierarchy for risk models was defined: from utopia to empirical data. J Clin Epidemiol. (2016) 74:167–76. doi: 10.1016/j.jclinepi.2015.12.005, PMID: 26772608

[76] Bogani G Pinelli C Chiappa V Martinelli F Lopez S Ditto A . Age-specific predictors of cervical dysplasia recurrence after primary conization: analysis of 3,212 women. J Gynecol Oncol. (2020) 31:e60. doi: 10.3802/jgo.2020.31.e60, PMID: 32808492 PMC7440983

[77] Hall MT Simms KT Lew JB Smith MA Brotherton JM Saville M . The projected timeframe until cervical cancer elimination in Australia: a modelling study. Lancet Public Health. (2019) 4:e19–27. doi: 10.1016/s2468-2667(18)30183-x, PMID: 30291040

[78] Canfell K . Towards the global elimination of cervical cancer. Papillomavirus Res (Amsterdam Netherlands). (2019) 8:100170. doi: 10.1016/j.pvr.2019.100170, PMID: 31176807 PMC6722296

[79] Gravitt PE Winer RL . Natural history of HPV infection across the lifespan: role of viral latency. Viruses. (2017) 9:267. doi: 10.3390/v9100267, PMID: 28934151 PMC5691619

[80] Zhou D Zhang D Wang Y Zhou K Tang W . Optimizing strategy for cervical cancer prevention in China: a comprehensive modeling analysis. Cost Effect Resour Alloc: C/E. (2025) 23:20. doi: 10.1186/s12962-025-00630-y, PMID: 40369618 PMC12080047

[81] Huang H Du C Liu G . China’s actions for post-political commitment to global strategy to accelerate cervical cancer elimination. Infect Agents Cancer. (2025) 20:77. doi: 10.1186/s13027-025-00708-5, PMID: 41163046 PMC12573873

